# The Influence of Blue Light and the BlsA Photoreceptor on the Oxidative Stress Resistance Mechanisms of *Acinetobacter baumannii*

**DOI:** 10.3389/fcimb.2022.856953

**Published:** 2022-03-24

**Authors:** Mariah S. Squire, Hope A. Townsend, Luis A. Actis

**Affiliations:** Department of Microbiology, Miami University, Oxford, OH, United States

**Keywords:** BlsA, blue light, oxidative stress response, catalase, superoxide dismutase

## Abstract

*Acinetobacter baumannii* is a catalase-positive Gram-negative bacterial pathogen that causes severe infections among compromised patients. Among its noteworthy regulatory mechanisms, this microorganism regulates its lifestyle through the blue light using flavin (BLUF) protein BlsA. This protein regulates a diverse set of cellular processes that include, but are not limited to, motility, biofilm formation, phenylacetic acid metabolism, iron uptake, and catalase activity. We set out to determine how *A. baumannii* regulates catalase activity and other related oxidative stress phenotypes in response to light. Notably, because *A. baumannii* ATCC 17978 encodes four catalase homologs – which we refer to as KatA, KatE, KatE2, and KatG – we also aimed to show which of these enzymes exhibit light- and BlsA-dependent activity. Our work not only provides insight into the general function of all four catalase homologs and the impact of light on these functions, but also directly identifies KatE as a BlsA-regulated enzyme. We further demonstrate that the regulation of KatE by BlsA is dependent on a lysine residue that we previously demonstrated to be necessary for the regulation of surface motility. Furthermore, we show that BlsA’s five most-C-terminal residues – previously considered dispensable for BlsA’s overall function – are necessary for the light-independent and light-dependent regulation of catalase and superoxide dismutase activities, respectively. We hypothesize that these identified critical residues are necessary for BlsA’s interaction with protein partners including the transcriptional regulators Fur and BfmR. Together these data expand the understanding regarding how *A. baumannii* uses light as a signal to control oxidative stress resistance mechanisms that are critical for its pathophysiology.

## Introduction

*Acinetobacter baumannii* is a highly ubiquitous opportunistic bacterial pathogen responsible for nosocomial and community-acquired infections and which causes significant disease in immunocompromised individuals (Whiteway et al., 2022). This pathogen is of major importance in clinical settings including burn units, long-term care facilities, and intensive care units. *A. baumannii* is the causative agent in a variety of serious disease outcomes including skin and soft-tissue infections, urinary tract infections, bacteremia, pneumonia, and meningitis (Peleg et al., 2008; Wong et al., 2017). Colonization often occurs in ventilated patients upon intubation, leading to ventilator-associated pneumonia, which carries high mortality rates. *A. baumannii* infections are difficult to treat due to the pathogen’s capacity to persist in medical environments, colonize the human host, and express antimicrobial resistance mechanisms, which has led to the emergence of multi-drug- and pan-drug-resistant strains (Lee et al., 2017; Wong et al., 2017; Moubareck and Halat, 2020). The high level of antimicrobial resistance observed in this pathogen can be attributed to a wide variety of β-lactamases (Lee et al., 2017), overexpression of efflux systems (Nowak et al., 2015), and downregulation of outer membrane proteins (Uppalapati et al., 2020). These factors highlight the importance of determining the molecular mechanisms by which *A. baumannii* influences its pathogenicity so that novel antimicrobial targets for future chemotherapeutic strategies can be identified.

Previous work in our laboratory has demonstrated that *A. baumannii* responds to environmental stimuli such as temperature and blue light through the blue light sensing protein, BlsA (*b*lue *l*ight *s*ensing *A*) (Mussi et al., 2010). BlsA, a relatively small (18.6 kDa) cytoplasmic protein, is an important regulator of a wide range of *A. baumannii* functions including biofilm formation and cell motility. This protein is classified as a blue light using flavin (BLUF) photoreceptor because of its canonical BLUF domain, which makes up two thirds of the molecule. The BLUF domain has a conserved structure comprising a five-stranded β-sheet flanked by two ⍺-helices and is responsible for the light-sensing functions of the protein (Brust et al., 2014; Wood et al., 2019). We refer to the remainder of the protein as BlsA’s “tail,” which is responsible for the regulatory functions imparted by *A. baumannii* in response to blue light (Wood et al., 2019). It was previously found that particular tail residues are critical for BlsA’s capacity to regulate motility in response to blue light. Specific lysine residues (K144 and K145) are indispensable for BlsA’s regulation of motility, whereas the protein’s last five amino acids are expendable for this regulation (Wood et al., 2019). The functional role and overall importance of BlsA’s last five amino acids remain elusive.

Because BlsA lacks canonical DNA-binding motifs and otherwise shares no sequence homology with known transcriptional regulators, it was initially hypothesized that this photoreceptor interacts with protein partners to accomplish its cellular regulation. Indeed, BlsA interacts with the ferric uptake regulator Fur (Tuttobene et al., 2018) and the acetoin catabolism negative regulator (Tuttobene et al., 2019) to moderate their regulation of gene expression under certain conditions. It is unclear whether the residues that are critical for BlsA’s regulation of motility are also necessary for its regulation through Fur, AcoN, and other unidentified effector proteins that would associate with BlsA. Additionally, in that regard, although BlsA is involved in the regulation of a long list of cellular processes (Muller et al., 2017), the full catalog of BlsA binding partners is still unknown.

Upon infection, bacterial pathogens like *A. baumannii* face key players of the innate immune response, including professional phagocytes like macrophages and neutrophils, which deploy an entire host of defense mechanisms including the production of reactive oxygen species (ROS) (Chen, 2020). In the lung, for example, *A. baumannii* infection leads to the recruitment of neutrophils that are necessary to control and contain the infection locally (van Faassen et al., 2007). Additionally, the ROS hydrogen peroxide (H_2_O_2_) is produced upon *A. baumannii* pulmonary infection (Juttukonda et al., 2019). After phagocytosis, the exposure of *A. baumannii* to ROS necessitates the production of counter-defense proteins. Enzymes like catalase, peroxidase, superoxide dismutase (SOD), and the universal stress protein A (UspA) enable *A. baumannii* to mitigate the harmful effects of ROS (Heindorf et al., 2014; Elhosseiny et al., 2015; Sun et al., 2016). Undoubtedly, even outside the context of infection, ROS defensive enzymes are essential for the survival of bacteria, including *Acinetobacter* species. For example, the polyextremophile environmental isolates *Acinetobacter* sp. Ver3 and sp. Ver7 obtained from Andean Lake Verde, a niche with high UV radiation and elevated salinity and heavy metal content, exhibit five-to-fifteen times as much catalase activity as the clinical isolate *A. baumannii* ATCC 19606^T^ (19606) (Di Capua et al., 2011). Rather than being necessary for protection within the context of an infection, such high catalase activity enables these strains to exhibit extreme resistance to H_2_O_2_ and other oxidative challenges they face in their environmental niche. Even within the hospital setting yet still outside of the context of infection, *A. baumannii* experiences ROS challenge in the form of disinfectants, which likely necessitates the deployment of oxidative stress resistance enzymes at temperatures lower than that in the human host (Lemmen et al., 2015).

*A. baumannii* encodes several catalase homologs, yet the types differ slightly strain-to-strain. Of particular note, the highly virulent and multi-drug-resistant (MDR) strain *A. baumannii* AB5075-UW (AB5075), encodes four catalase homologs: KatA, KatE, KatG, and KatX, and the significance of each of these proteins with regard to H_2_O_2_ degradation and resistance has been thoroughly investigated (Sun et al., 2016). KatE is a large subunit monofunctional hydroperoxidase type II (HPII) catalase without peroxidase activity and is responsible for the main catalase activity in the AB5075 strain. It is also more readily produced in the stationary phase of growth. On the other hand, KatG is a hydroperoxidase type I (HPI) catalase-peroxidase and plays a significant role in resistance to H_2_O_2_. KatA and KatX, although annotated as a small subunit monofunctional catalase and small catalase domain-containing protein, respectively, do not play a detectable role in the degradation of or resistance to H_2_O_2_ (Sun et al., 2016). In the 19606 strain, both the expression of *katE* and catalase activity of two unidentified proteins are controlled by BlsA in response to light (Muller et al., 2017). However, the implications of this light-dependent regulation on the physiology of *A. baumannii*, the importance of BlsA’s key tail residues in this regulation, and the influence of light on other oxidative stress resistance enzymes in *A. baumannii* remains largely unknown.

Encoded in the clinical isolate *A. baumannii* ATCC 17978 (17978) are four putative catalase genes: ACX60_16060, ACX60_11390, ACX60_11205, and ACX60_00500. Because of their sequence similarities to the AB5075 homologs noted above and more fully described below, we have adopted the same notation used in the AB5075 strain and, for the sake of clarity henceforth, will refer to the proteins encoded by the 17978 strain genes ACX60_11390, ACX60_11205, and ACX60_16060 as KatA, KatE, and KatG, respectively. We will refer to the final homolog (encoded at locus ACX60_00500) as KatE2 because of its relatively high sequence identity (~80%) to the recently described HPII ^AV3^KatE2 from *Acinetobacter* sp. Ver3. Notably, ^AV3^KatE2 and a similar catalase in the same extremophile, ^AV3^KatE1, play important roles in the response to H_2_O_2_ challenge and UV exposure (Sartorio et al., 2020). Recently, the role of KatE2 with regard to the OxyR-mediated response to H_2_O_2_ has been investigated in the 17978 strain (Juttukonda et al., 2019). It is worth noting that in said study, this particular homolog was referred to as KatE.

In this work, we set out to examine the light- and BlsA-dependence of select oxidative stress resistance mechanisms and to analyze the involvement of selected BlsA tail residues in the regulation of catalase activity in the 17978 strain. We chose to examine these processes in the 17978 strain, in particular, because it is in this strain that the selected BlsA tail residues have been characterized in terms of relative importance for BlsA’s regulation of surface-associated motility in response to light (Wood et al., 2019). Additionally, with a tested murine model, we consider that future studies involving the 17978 strain would provide a more robust understanding of the diverse pathological strategies of *Acinetobacter baumannii* beyond the AB5075 strain.

Through an analysis of BlsA tail residue mutants as well as individual catalase mutants, we determined that one of BlsA’s lysine residues, which we recently described as necessary for the regulation of surface-associated motility, is also critical for the regulation of the catalase homolog, KatE. Additionally, we determined that BlsA’s last five residues – which are dispensable for its regulation of surface-associated motility – play a role in the regulation of KatE in a light-independent manner and in the regulation of SOD activity in a light-dependent manner. These data provide an expanded understanding of the molecular mechanisms involved in BlsA’s global regulatory functions and a deepened understanding of oxidative stress resistance mechanisms in *A. baumannii*. Finally, we present evidence supporting the potentially novel role of the BfmR transcriptional regulator in the light-dependent BlsA-mediated regulation of KatE and the overall catalase activity expressed by the 17978 strain.

## Materials and Methods

### Bacterial Strains and Culture Conditions

All strains and plasmids used in this study are listed in **Table 1**. A*. baumannii* strains were routinely cultured overnight (12-16 h) at 37°C in Luria Bertani (LB) broth or on LB agar plates and supplemented with antibiotics when necessary. For all biological assays (catalase, zymogram, peroxide resistance, SOD, and bacterial growth assays), swimming broth (SB) comprising 1% tryptone and 0.5% NaCl was used as the culturing medium. When necessary, SB was supplemented with 1.5% (w/v) agar to yield swimming agar (SA). For blue light treatments during bacterial culturing, a blue light LED array (peak emission wavelength, 469 nm) was used as described previously (Mussi et al., 2010; Wood et al., 2019).

**Table 1:** Bacterial strains and plasmids used in this work.

Strain/plasmid	Relevant characteristic(s)^a^	Source/reference
*A. baumannii*		
ATCC 17978 (17978)	Clinical isolate	ATCC
17978.OR	*blsA*::*aph* derivative of ATCC 17978; Km^R^	Mussi et al., 2010
17978.OR.WT	*blsA*::*aph* derivative of ATCC 17978 harboring pMU1202; Km^R^; Zeo^R^	Wood et al., 2019
17978.OR.K144E	*blsA*::*aph* derivative of ATCC 17978 harboring pMU1276; Km^R^; Zeo^R^	Wood et al., 2019
17978.OR.K145E	*blsA*::*aph* derivative of ATCC 17978 harboring pMU1250; Km^R^; Zeo^R^	Wood et al., 2019
17978.OR.Δ5	*blsA*::*aph* derivative of ATCC 17978 harboring pMU1232; Km^R^; Zeo^R^	Wood et al., 2019
17978*.katA*	*katA*::*ermAM* derivative of ATCC 17978; Em^R^	This study
17978.*katE*	*katE*::*ermAM* derivative of ATCC 17978; Em^R^	This study
17978.*katE*2	*katE2*::*ermAM* derivative of ATCC 17978; Em^R^	This study
17978.*katG*	*katG*::*ermAM* derivative of ATCC 17978; Em^R^	This study
17978.*katE*.WT	*katE::ermAM der*ivative of ATCC 17978 harboring pMU1336; Em^R^, Km^R^, Zeo^R^	This study
17978.*katE2*.WT	*katE2::ermAM* derivative of ATCC 17978 harboring pMU1338; Em^R^, Km^R^, Zeo^R^	This study
19606^T^ (19606)	Clinical isolate, type strain	ATCC
19606.*bfmR*	*bfmR*::EZ::TN<R6K*γori*/KAN-2> derivative of 19606^T^	Tomaras et al., 2008
*E. coli*		
Top10	DNA recombinant processes	Invitrogen
Plasmids		
pAT04	Mutagenesis plasmid, Tet^R^	Tucker et al., 2014
pCR Blunt II-TOPO	Cloning vector; Km^R,^Zeo^R^	Invitrogen
pCR8 TOPO TA	Cloning vector; Sp^R^	Invitrogen
pMU368	*E. coli*-*A. baumannii* shuttle vector; Km^R^, Zeo^R^	Dorsey et al., 2006
pMU1202	pMU368 harboring *blsA* and its predicted promoter sequence from 17978; Km^R^, Zeo^R^	Wood et al., 2019
pMU1232	pMU368 harboring *blsA:Δ5* and its predicted promoter sequence from 17978; Km^R^, Zeo^R^	Wood et al., 2019
pMU1250	pMU368 harboring *blsA:K145E* and its predicted promoter sequence from 17978; Km^R^, Zeo^R^	Wood et al., 2019
pMU1276	pMU368 harboring *blsA:K144E* and its predicted promoter sequence from 17978; Km^R^, Zeo^R^	Wood et al., 2019
pMU1336	pMU368 harboring *katE* and its predicted promoter sequence from 17978; Km^R^, Zeo^R^	This work
pMU1338	pMU368 harboring *katE2* and its predicted promoter sequence from 17978; Km^R^, Zeo^R^	This work

a Km^R^, kanamycin resistance; Zeo^R^, zeocin resistance; Em^R^, erythromycin resistance; Sp^R^, spectinomycin resistance; Tet^R^, tetracycline resistance.

### DNA Procedures

Plasmid DNA was routinely isolated from *E. coli* using a commercial kit (Epoch Life Science). *A. baumannii* genomic DNA (gDNA) was isolated as previously described (Barcak et al., 1991). DNA polymerases, restriction enzymes, and T4 DNA ligase were purchased from New England Biolabs and were used according to the manufacturer’s instructions. All primers used in this study, which were either custom designed or based on sequences provided by cloning kits (**Table 2**), were purchased from IDT. The pCR Blunt II-TOPO and pCR8/GW/TOPO TA cloning kits were acquired from Invitrogen and used according to the manufacturer’s instructions. All DNA sequencing was carried out using automated DNA sequencing (BigDye, Applied Biosystems).

**Table 2:** Primers used in this study.

Primer	Target Gene	Primer Sequence
4100	*ermAM* cassette	GCAAACTTAAGAGTGTGTTG
4101	*ermAM* cassette	CCTTTAGTAACGTGTAACTTTC
4513	*csuAB* (gDNA screen)	GGTCATTCGACCTAATTAATGG
4514	*csuAB* (gDNA screen)	GCTGCAAGAAGTGATTTCTGAATG
4685	*katE* (RT-qPCR)	GCTGGTATGGGATGAAGCTC
4686	*katE* (RT-qPCR)	GGTCCAGCAAATCGAAGTCA
4744	*katE2* (cloning, screening)	ATGTGAGATATCCTATGCTTCAACTTCCAG
4745	*katE2* (cloning, screening)	TGATATGATATCGGCTCTATCTATTGCAATTG
4746	*katE2* (iPCR)	GATCTGAAAATGGTGCAAG
4747	*katE2* (iPCR)	TGCATACACTGTGCTTAG
4750	*katE* (iPCR)	GAGTTCCAGCATGTTAC
4751	*katE* (iPCR)	GTCGTTCATGTCCATTAC
4752	*katA* (cloning, screening)	ATGTGAGAATTCCGCCTGATAAACTTATCGAG
4753	*katA* (cloning, screening)	TGATATGAATTCCTTTAGCAGTCATGCTTG
4754	*katA* (iPCR)	GGATTTGGCAAAGCAAC
4755	*katA* (iPCR)	TGCGATTACAGCAATACC
4756	*katG* (cloning, screening)	ATGTGAGAATTCCTGCTTGATGATGTCATG
4757	*katG* (cloning, screening)	TGATATGAATTCGCTGCATAAGAAGTTTGC
4758	*katG* (iPCR)	CTTGACCGTTTTGACTTAG
4759	*katG* (iPCR)	AGGACATTTTGATTCGTTTG
4760	*katE* (cloning, screening)	ATGTGAGAATTCCATAACGAAACCAGACAG
4761	*katE* (cloning, screening)	TGATATGAATTCGGTTCTACAGCATTTTGG
4762	*katG* (mutagenesis)	GATATGGTTAAATCTAACCG
4763	*katG* (mutagenesis)	AGGAAATTCCTTATCACG
4764	*katA* (mutagenesis)	TGATCAAGATAGTACAGC
4765	*katA* (mutagenesis)	GATAGATGACCAATCACG
4766	*katE* (mutagenesis)	CACTCATTTAACTGAACAC
4767	*katE* (mutagenesis)	CCATTCCTTATATGGAGG
4768	*katE2* (mutagenesis)	AGCCATTAAAGTGAACTG
4769	*katE2* (mutagenesis)	CATTAATGCAGTATGTCC
4792	*katE* (complementation)	ATGTGAGGATCCCAAACTTACTCCGTTTCTTG
4793	*katE* (complementation)	TGATATGGATCCCTTGCCTCAGATGATG
4794	*katE2* (complementation)	ATGTGAGGATCCCAGTAGTCTAGCAGAATTC
4795	*katE2* (complementation)	TGATATGGATCCGATTGAGGTTACAGGAC
4796	*recA* (RT-qPCR)	TGCACCATTTGTGCCTGTAG
4797	*recA* (RT-qPCR)	TACAGAAAGCTGGTGCATGG
M13F	N/A	GTAAAACGACGGCCAG
M13R	N/A	CAGGAAACAGCTATGAC

Underlined nucleotides indicate BamHI restriction sites. “iPCR” primers were used in inverse PCR reactions to generate gene deletions; “mutagenesis” primers were used to amplify interrupted alleles to use in homologous recombination experiments; “complementation” primers were used in the generation of complementing plasmids.

### Generation of Catalase Mutants

Catalase mutants were generated based on a recombineering approach designed for *A. baumannii* (Tucker et al., 2014). Briefly, each gene along with at least 500 nucleotides up- and downstream of each coding region was PCR amplified using Q5 DNA polymerase, 17978 wildtype gDNA, and the following primer sets: 4752 and 4753 for *katA*; 4760 and 4761 for *katE*; 4744 and 4745 for *katE2*; and 4756 and 4757 for *katG* (**Table 2**). The amplicons were cloned into pCR-Blunt. After successful cloning was verified by PCR screening using M13 primers, each gene was then subjected to a deletion-insertion mutation that removed more than 90% of each coding region. Deletions were generated by performing inverse PCR using the following primer pairs: 4754 and 4755 for *katA*; 4750 and 4751 for *katE*; 4746 and 4747 for *katE2*; and 4758 and 4759 for *katG* (**Table 2**). For selection purposes, in place of each deletion was ligated the *Lactococcus lactis* pIL252 *ermAM* gene encoding erythromycin resistance with its promoter region as described before (Fiester et al., 2016). After confirmation by PCR and automated DNA sequencing, each mutated gene was PCR amplified using Q5 DNA polymerase and the following primer sets: 4764 and 4765 for *katA*; 4766 and 4767 for *katE*; 4768 and 4769 for *katE2*; and 4762 and 4763 for *katG* (**Table 2**). Clean PCR products were used to transform 100 µL aliquots of electrocompetent 17978 cells carrying the pAT04 plasmid, prepared as described previously (Tucker et al., 2014). Electroporation of 1-12 µg PCR product was carried out at 2,500 V as described before (Dorsey et al., 2002), after which bacteria were allowed to recover for four h in SOC medium supplemented with 2 mM IPTG for induction of gene expression from pAT04. After recovery, bacteria were pelleted, and recombinants were selected for on LB agar containing 20 µg/mL erythromycin. Resulting colonies were screened *via* PCR using primers annealing outside homologous regions to confirm successful gene deletion and insertion. Primers used are numbered as follows in **Table 2**: 4752 and 4753 for the *katA* interruption; 4760 and 4761 for the *katE* interruption; 4744 and 4745 for the *katE2* interruption; and 4756 and 4757 for the *katG* interruption. Mutations were finally verified by automated DNA sequencing.

### Gene Complementation

Catalase genes and their predicted promoters, which were predicted with the aid of Softberry BPROM software (Solovyev and Salamov, 2011), were amplified using primers with 5’ *Bam*HI restriction sites as follows: 4792 and 4793 for *katE*; 4794 and 4795 for *katE2*. (**Table 2**). After amplification, PCR products were cloned into pCR8, and the resulting plasmids (**Table 1**) were used to transform Top10 *E. coli*. Positive transformants were selected for on LB agar containing 100 µg/mL spectinomycin. Genes and predicted promoters were then subcloned from pCR8 into the *E. coli-A. baumannii* shuttle vector pMU368 (**Table 1**) by restriction cloning using *Bam*HI. Resulting plasmids (**Table 1**) were used to transform Top10 *E. coli* competent cells according to the manufacturer’s instructions. Positive transformants were selected for on LB agar supplemented with 50 µg/mL kanamycin (Km). Successful cloning of each gene with its predicted promoter into pMU368 was confirmed by PCR screening using M13 primers and automated DNA sequencing. Complementing plasmids were introduced into respective electrocompetent *A. baumannii* mutant strains by electroporation (Dorsey et al., 2002); positive transformants were selected for on LB agar containing 50 µg/mL Km. The presence of complementing plasmids as independent replicons without detectable rearrangement was confirmed by isolating plasmids from *A. baumannii* derivatives using the hot-Triton method (Actis et al., 1993). Isolated plasmids were then transformed into *E. coli* Top10 competent cells, and Km resistant colonies were used to isolate plasmids that were examined by restriction analysis (Sambrook and Russell, 2001).

### Transcriptional Analysis

Overnight bacterial cultures were diluted 1:100 in SB and grown at 24°C in darkness with shaking at 150 rpm to an OD_600_ of approximately 0.8. Bacteria in 1 ml of culture were pelleted by centrifugation and then lysed using the TRIzol reagent (Invitrogen). Total RNA was extracted using the Zymo Direct-zol RNA Miniprep Plus kit (Zymo Research) according to the manufacturer’s instructions. DNA was removed from RNA samples using the TURBO DNA-free kit (Invitrogen) according to the manufacturer’s instructions, after which samples were verified to be DNA-free using Taq DNA polymerase (New England Biolabs) and primers 4513 and 4514 (**Table 2**) for the detection of *csuAB* as an indicator of gDNA contamination. Total RNA was reverse transcribed using the iSCRIPT cDNA synthesis kit (Bio-Rad) according to the manufacturer’s instructions. Relative gene transcription was measured by qPCR using the iTaq Universal SYBR Green supermix (Bio-Rad) according to the manufacturer’s instructions and as previously described (Fiester et al., 2016). Primers 4685 and 4686 (**Table 2**) were used to measure the expression of *katE.* Expression of *katE* was normalized to that of the constitutively expressed reference gene *recA*, which was measured using primers 4796 and 4797 (**Table 2**). qPCR efficiencies were at least ~90% in all reactions. Transcriptional analysis was performed in this way for each strain using two biological replicates, the first analyzed in technical duplicate and the second analyzed in technical triplicate (*n* = 5).

### Catalase Activity Assay

Catalase activity was measured using an assay as previously described (Iwase et al., 2013). In brief, overnight bacterial cultures were diluted 1:100 in SB and then incubated at 24°C in darkness or under blue light illumination provided by a LED array as described above with shaking at 150 rpm to an OD_600_ of approximately 0.8. Culture samples representing equal numbers of bacteria were then pelleted by centrifugation, washed in sterile phosphate-buffered saline (PBS), and resuspended in equal volumes of PBS. Catalase activity was assayed by mixing 100 μL bacteria prepared as described above with 100 μL 1% Triton X-100 and 100 μL 30% H_2_O_2_ (Fisher Chemical) in a glass culture tube. Total foam formation was measured 15 min after mixing and used as a proxy for catalase activity. The catalase activity of each bacterial strain in each condition (blue light treatment or darkness) was measured using three independent biological replicates, each in technical triplicate (*n* = 9).

### Zymogram Experiments

For zymogram analysis, overnight bacterial cultures were diluted 1:100 in SB and then incubated at 24°C in darkness or under blue light with shaking to an OD_600_ of approximately 0.8. Equal volumes of bacteria were collected by centrifugation at 6,000 x *g* for 10 min, resuspended in extraction buffer (50 mM Tris-HCl, pH 8.0), and were then lysed by bead beating. Lysates were clarified by centrifugation at 12,000 x *g* for 20 min, and protein concentration of the resulting supernatant was measured using the Bradford method (Bradford, 1976) using a commercial kit (Bio-Rad). Equal amounts of protein were loaded onto a 10% non-denaturing polyacrylamide gel and were run under non-denaturing conditions. In-gel catalase activity was visualized as previously described (Gamero-Sandemetrio et al., 2017). This analysis was carried out twice using separate biological replicates each time.

### Disc Diffusion Assay

Resistance to H_2_O_2_ was analyzed by performing disc diffusion assays as described previously (Juttukonda et al., 2019) with some modifications. Briefly, bacterial cultures were grown at 24°C for 18 h with shaking. Then, 200 μL of bacterial culture were added to 20 mL warm SA, and equal volumes of this mixture were aliquoted into two separate 60-mm Petri dishes. After drying for 10 min, 6-mm sterile filter discs were deposited on the top of the agar, and 10 μL 9.8 M H_2_O_2_ was added to each disc. Plates were incubated overnight at 24°C in darkness or under blue light, and diameters of growth inhibition were measured. Disc diffusion experiments were carried out for each tested bacterial strain in each condition using three independent biological replicates (*n* = 3).

### Growth Analysis

Bacterial growth in SB only or in the same medium supplemented with H_2_O_2_ was analyzed by monitoring OD_600_ values over time. Overnight liquid bacterial cultures were diluted 1:100 in SB supplemented with 0 or 2.5 mM H_2_O_2_ and were grown with shaking and in the presence of blue light or in darkness, as indicated, at 24°C. OD_600_ was measured using a spectrophotometer. All growth experiments were carried out at least twice using independent biological replicates each time.

### SOD Assays

SOD activity was measured in bacteria cultured as described for catalase activity analysis. After culturing, cells were pelleted by centrifugation (6 min at 6,000 x *g*), washed in PBS, and lysed by sonication using an ultrasonic cell disruptor with a microtip (Fisherbrand). Lysed samples were centrifuged for 6 min at 6,000 x *g* to collect cell debris, and clarified samples were assayed for SOD activity using the commercial SOD Assay Kit-WST (Dojindo Molecular Technologies Inc.) according to the manufacturer’s instructions. SOD activity was normalized to total protein concentration as measured by a Bradford assay. For each tested strain/condition, a total of 2-3 independent biological replicates in technical triplicate were analyzed.

### Bioinformatic Analysis

Predicted amino acid sequences of selected proteins were downloaded from NCBI. Sequence alignments and comparisons were performed using MUSCLE (Edgar, 2004). Binding and active sites of amino acid sequences were identified using Swiss-Prot or TrEMBL sections of UniProt (UniProt Consortium, 2021). A phylogenetic tree was constructed based on a comparative genomic analysis of the catalase homologs from *A. baumannii* strains 17978, 19606, AB5075, and *Acinetobacter* sp. Ver3. Multiple alignment of the amino acid sequences of all catalase homologs was carried out using MUSCLE, implemented within the Molecular Evolutionary Genetics Analysis tool, MEGA version X (Kumar et al., 2008; Stecher et al., 2020). This tool was also used to infer protein phylogeny using the Maximum Likelihood method based on an initial tree constructed from the Neighbor Joining method. Evolutionary distances were computed using the Poisson correction method (Zuckerkandl and Pauling, 1965). A discrete Gamma distribution was used to model evolutionary rate differences among sites [5 categories (+G, parameter - 200.0000)]. Reliability of the inferred tree was tested by bootstrapping with 800 repetitions.

### Statistical Analysis

The statistical significance of the experimental data sets was analyzed using GraphPad Prism version 9.0 for MacOS with the appropriate statistical test applied for each experiment as described in the figure legends. *P* values ≤ 0.05 were considered statistically significant in these analyses. Error bars represent the standard deviation of the mean of each data set.

## Results

### Investigation of the Light Dependence of Catalase Functions

**Figure 1:**
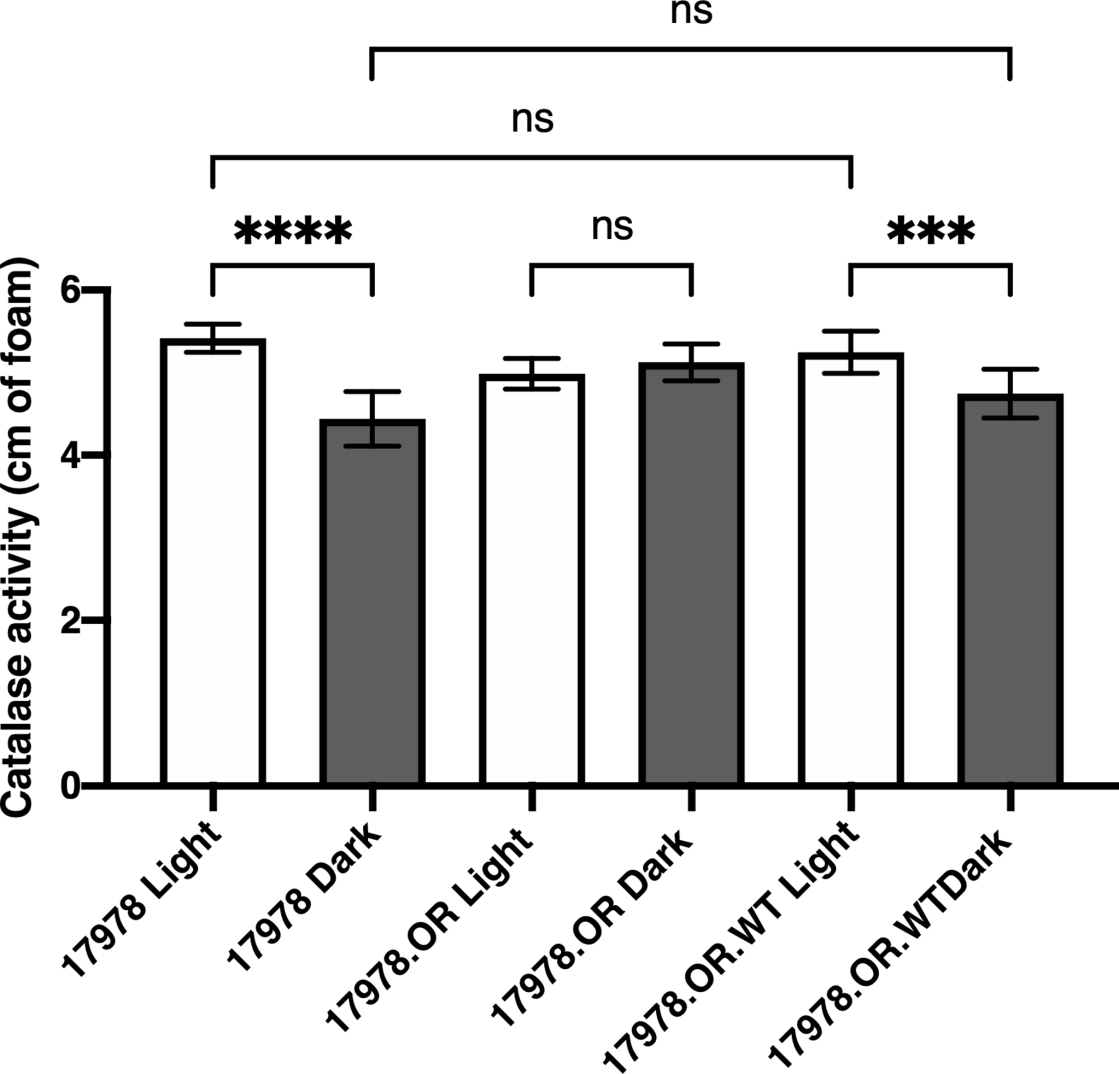
Light- and BlsA-dependence of catalase activity. Catalase activity of the wildtype 17978 strain, the isogenic OR *blsA^-^* mutant (17978.OR), and the isogenic *blsA^-^* mutant complemented with a pMU368 derivative encoding wildtype BlsA (17978.OR.WT) was measured using culture samples representing equal numbers of bacteria that had been grown in SB under blue light or in darkness with shaking to exponential phase at 24°C. The catalase activity of each strain was analyzed using three independent biological replicates in technical triplicate (*n* = 9). Error bars represent the standard deviations of the data sets. Horizontal bars with symbols indicate results of ordinary one-way ANOVA with Sidak’s multiple comparisons *post-hoc* test (***P ≤ 0.001; ****P ≤ 0.0001; ns, not significantly different).

In the 19606 strain, BlsA is responsible for the regulation of catalase gene expression and activity in response to blue light (Muller et al., 2017). These observations prompted us to determine whether the same phenomenon is observed in the 17978 isolate and, if so, whether the BlsA residues that are necessary for the regulation of bacterial surface-associated motility, another BlsA- and light-dependent phenotype, are critical for the regulation of catalase activity. Notably, using a different means of measurement, we were able to demonstrate that catalase activity is a light-regulated activity in 17978 (**Figure 1**). Although there is indeed catalase activity under both conditions, this activity is regulated by light such that there is significantly higher activity when wildtype 17978 bacteria are cultured under blue light compared to in darkness. This regulation is dependent on the BlsA photoreceptor: in the isogenic *blsA*::*aph* derivative (17978.OR), light regulation is abolished, yet when this mutant is complemented in *trans* with the wildtype *blsA* allele (17978.OR.WT), the light-dependent phenotype is restored.

The genome of the 17978 strain harbors genes encoding four catalase homologs: KatA, KatE, KatE2, and KatG. All homologs include the critical residues necessary for catalase or peroxidase activity including binding sites for heme, enzymatic active sites, and metal binding sites (**Supplementary Figures 1**, **2**). Notably, each catalase homolog of the 17978 strain shares above 99% identity with and similarity to cognate catalase homologs in both the 19606 and AB5075 strains, with the exception of KatE2, which is absent in the AB5075 strain (**Table 3**). More specific information about which catalase homologs and associated physiological functions are regulated by light and/or BlsA and the mechanism of said regulation is needed. This conclusion is supported by the recent report by Muller et al. (2017) that identified two proteins expressing light- and BlsA-dependent catalase activity, although their RNA-Seq data identified only one gene being differentially transcribed in response to illumination (Muller et al., 2017). Likewise, although no light-mediated regulation of catalase activity has been explored in the AB5075 strain, the role of different catalase homologs has been significantly characterized in this strain (Sun et al., 2016). Importantly, the AB5075 strain also encodes a BlsA-like homolog whose light sensing and regulatory functions remain to be determined (Gallagher et al., 2015).

**Table 3:** Catalase homologs in the *A. baumannii* strains ATCC 17978, ATCC 19606^T^, and AB5075. The format includes name (Protein ID), annotated function, and length.

ATCC 17978	ATCC 19606^T^*	AB5075
**KatE** (AKQ27286.1)	**KatE** (BCA99837.1)	**KatE** (AKA32163.1)
HPII	catalase/HPII	catalase/HPII
712	713	712
**KatA** (AKQ27323.1)	**KatA** (BCA99902.1)	**KatA** (AKA32231.1)
catalase (spA like)	catalase-related peroxidase	catalase (spA like)
354	338	354
**KatE2** (AKQ25274.1)	**KatE2** (BCA97756.1)	NA
catalase (clade 1)	catalase (clade 1)	
507	507	
**KatG** (AKQ28202.1)	**KatG** (BCB01069.1)	**KatG** (AKA33165.1)
peroxidase	catalase peroxidase	catalase/peroxidase HPI
718	718	718

*ATCC 19606^T^ genome consists of only ab initio annotations.

**Figure 2:**
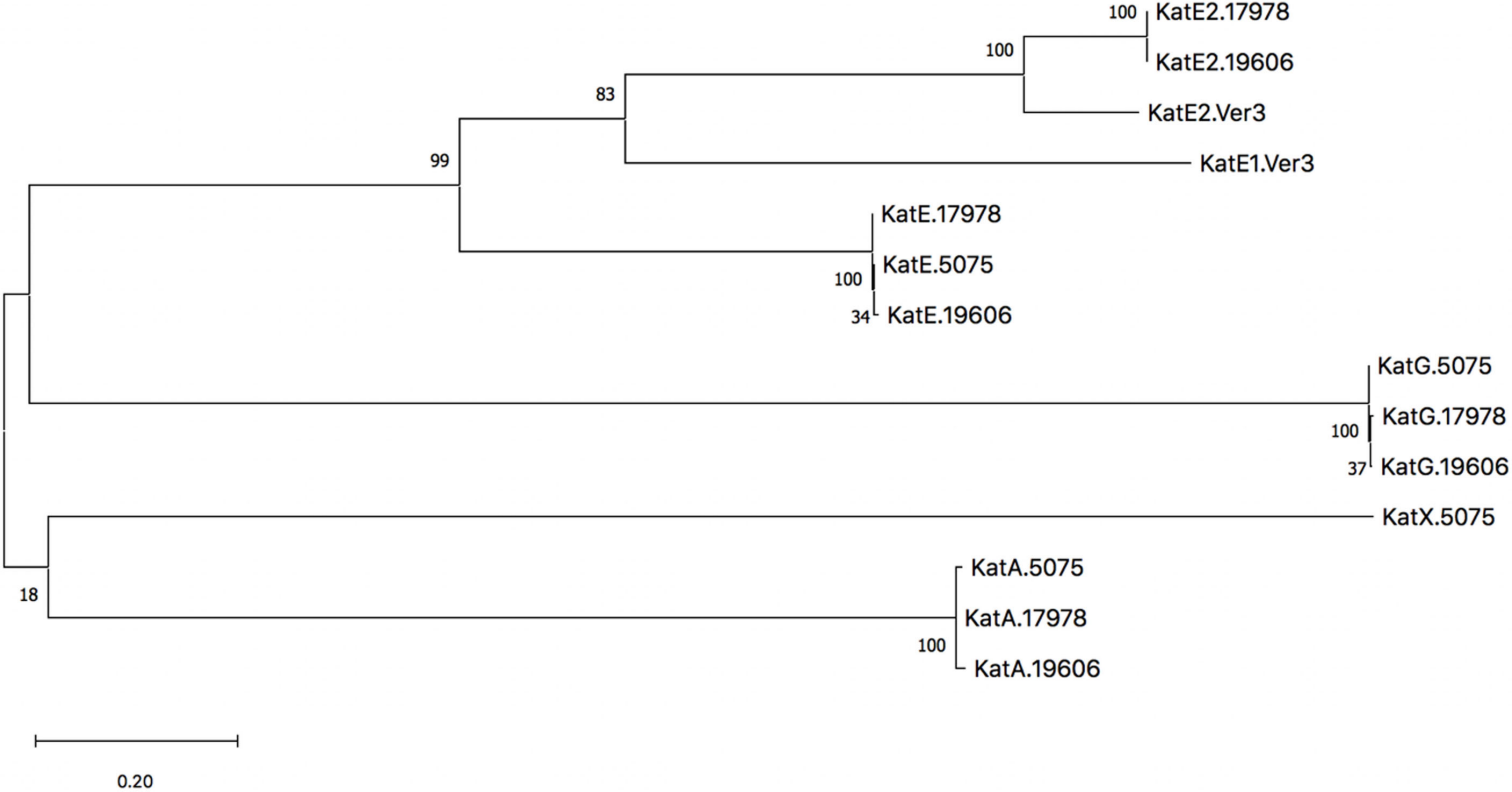
Phylogenetic tree of catalases from *A. baumannii* strains ATCC 17978, ATCC 19606^T^, AB5075, and *Acinetobacter* sp. Ver3. The dendrogram was constructed with mega version X using the Maximum Likelihood method based on an initial tree constructed from the Neighbor Joining method, and the robustness of the major branching points is indicated by the bootstrap values (800 repetitions). The tree with the highest log likelihood is shown and branch lengths are measured in the number of substitutions per site.

Despite similarities between strains of *A. baumannii*, within each strain the catalase homologs are distinct in sequence and annotated function. In the 17978 strain specifically, KatE and KatE2 have the greatest similarity and identity to each other with values of 40.5% and 27.3%, respectively. The second highest similarity and identity are shared between KatA and KatE2 with similarity and identity values of 28.7% and 19.1%, respectively. Although there are significant amino acid sequence differences among the homologs, KatA, KatE, and KatE2 all possess heme-binding domains (**Supplementary Figure 1**), whereas KatG contains a domain belonging to the PRK15061 superfamily that confers both catalase and peroxidase activities (**Supplementary Figure 2**). Characterization of KatE and KatG has been particularly explored in the AB5075 isolate as previously noted (Sun et al., 2016). However, unlike the 17978 and 19606 strains, the AB5075 strain does not possess a KatE2 homolog. Notably, a clue into the significance of KatE2 with regard to the physiology of bacteria that possess this type of catalase may be taken from *Acinetobacter* sp. Ver3, which encodes two catalases, ^AV3^KatE1 and ^AV3^KatE2, that are both highly similar to 17978’s KatE2 (**Figure 2**). Interestingly, the ^AV3^KatE1 and ^AV3^KatE2 catalases are implicated in UV survival and H_2_O_2_ resistance in this polyextremophile (Sartorio et al., 2020). The phylogenetic relationships of the catalase homologs from these four strains are illustrated by the tree shown in **Figure 2**. The distinctive functions among these clades of catalase homologs indicates that the pathophysiological impact of light-mediated catalase regulation in *A. baumannii* is dependent on the catalase homolog being regulated.

**Figure 3:**
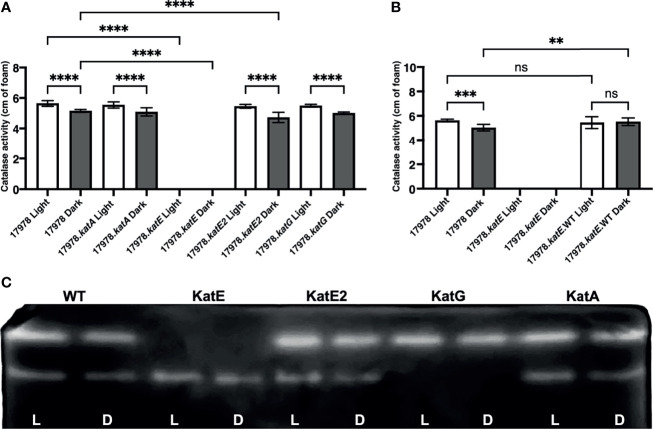
The effect of light on catalase activity of 17978 strains. Light-dependent catalase regulation in the wildtype 17978 and isogenic catalase mutant strains. **(A)** Catalase activity of 17978 and *katA*^-^, *katE^-^, katE2^-^*, and *katG*^-^ mutant strains (17978.*katA*, 17978.*katE*, 17978.*katE2*, and 17978.*katG*, respectively) was measured using equal numbers of bacteria that had been grown in SB under blue light or in darkness with shaking to exponential phase at 24°C. **(B)** Catalase activity of the complemented *katE^-^* strain (17978.*katE*.WT) was compared to wildtype and the isogenic *katE*^-^ mutant using the same conditions described in **(A)** The catalase activity of each strain was analyzed using three independent biological replicates in technical triplicate (*n* = 9). Error bars represent the standard deviations of the data sets. Horizontal bars with symbols indicate results of ordinary one-way ANOVA with Sidak’s multiple comparisons *post-hoc* test (**P ≤ 0.01; ***P ≤ 0.001; ****P ≤ 0.0001; ns, not significantly different). Only statistically different values are identified in the graph in panel (**A**). (**C**) Zymogram image displaying in-gel catalase activity after performing native-PAGE of equal amounts of the soluble protein from wildtype bacteria and the four *kat* mutants. Catalase activity was visualized after in-gel incubation with H_2_O_2_ and staining with a 2% ferric chloride and 2% potassium ferric cyanide solution.

Because 17978 cells regulate catalase activity in response to light but encode several candidate homologs, we aimed to determine which of these homologs are indeed responsible for the activity observed under our experimental conditions, i.e., which are regulated by light through BlsA. For this purpose, deletion-insertion mutations were generated in all four catalase genes using a recombineering mutagenesis approach developed specifically for *A. baumannii* (Tucker et al., 2014). Importantly, none of these mutations resulted in a significant growth deficiency compared to wildtype as well as among themselves (**Supplementary Figure 3**). The same assay described above was then used to test the catalase activity of each mutant cultured to exponential phase in either blue light or darkness. Compared to wildtype 17978 cells, mutations of *katA* and *katG* caused no significant decrease in catalase activity in mutant bacteria (17978.*katA* and 17978.*katG*, respectively) that had been cultured in either condition (**Figure 3A**). Regarding the *katE2* mutation, the catalase activity of this strain (17978.*katE2*) was decreased significantly compared to wildtype only when bacteria had been cultured in darkness (**Figure 3A**). Nonetheless, in the absence of any of these three genes, catalase activity was still regulated by light. On the other hand, the *katE* deletion mutant strain (17978.*katE*) exhibited a complete absence of catalase activity when bacteria had been cultured in either condition (**Figure 3A**). Notably, we were able to complement the activity of 17978.*katE* to wildtype levels, albeit without the light-dependent differential response, when *katE* was expressed from a complementing plasmid (**Figure 3B**). To provide additional evidence that it is indeed KatE that BlsA is regulating in response to blue light, we carried out zymogram analyses of 17978 wildtype cells and cells of the four catalase mutant strains. According to the results as shown in **Figure 3C**, the analysis of soluble proteins isolated from 17978 cells under non-denaturing conditions revealed two bands representing proteins that decomposed H_2_O_2_, and both bands were slightly brighter in bacteria that had been cultured under blue light compared to in darkness. The soluble protein profile of the 17978.*katE* strain lacked the top band, indicating that the light-regulated catalase is indeed KatE. Notably, in the protein profile of the 17978.*katG* strain, the bottom band was lacking, indicating that this band represents KatG. Importantly, in neither the 17978.*katE2* nor the 17978.*katA* strains were there noticeable differences in the protein profile compared to the wildtype 17978 strain (**Figure 3C**).

**Figure 4:**
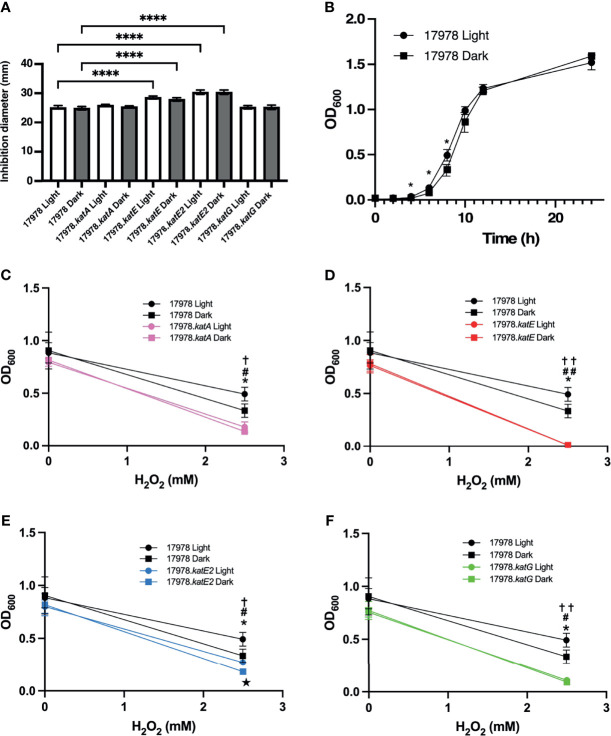
The effect of light on peroxide resistance of 17978 catalase mutants. The impact of blue light on the peroxide resistance of the 17978 wildtype and isogenic catalase mutants grown at 24°C was measured by two methods. **(A)** The resistance of wildtype and catalase mutant strains under blue light or in darkness was analyzed using H_2_O_2_ disc diffusion analysis. The averages of diameters of inhibition from three independent experiments are shown (*n* = 3), and error bars represent the standard deviations of the data sets. Horizontal bars with symbols indicate results of ordinary one-way ANOVA with Sidak’s multiple comparisons *post-hoc* test (****P ≤ 0.0001). **(B)** 17978 wildtype bacteria were grown under blue light or in darkness in SB supplemented with 2.5 mM H_2_O_2_ at 24°C with shaking, and optical density was measured over time. Error bars represent the standard deviation of each data set. Horizontal bars with symbols indicate results of unpaired *t* test analysis (*P ≤ 0.05). **(C–F)** Growth of catalase mutants compared to wildtype bacteria at 8 h when grown in SB or SB supplemented with 2.5 mM H_2_O_2_ under blue light or in darkness. Growth data are the average of at least two individual growth experiments for each strain, and error bars represent the standard deviations of the data sets. Horizontal bars and symbols indicate results of unpaired *t* test analysis (*P ≤ 0.05 between 17978 Light and Dark; **✝***P* ≤ 0.05 between 17978 Light and respective *kat* mutant Light; **#***P* ≤ 0.05 between 17978 Dark and respective *kat* mutant Dark; ★*P* ≤ 0.05 between respective *kat* mutant Light and Dark; **✝✝***P* ≤ 0.01 between 17978 Light and respective *kat* mutant Light; **##***P* ≤ 0.01 between 17978 Dark and respective *kat* mutant Dark). Only statistically significant differences are noted on the graphs. Full growth curve data are shown in **Supplementary Figure 5**.

In a previous study of the 19606 strain, it has been proposed that BlsA is responsible for the light-dependent differential production of two different catalases, which were predicted to be KatE and KatG (Muller et al., 2017). Because of this previous hypothesis and because KatG is a catalase-peroxidase, we investigated whether peroxidase activity is also regulated in response to blue light. Peroxidase activity is related to H_2_O_2_ resistance based on the hypothesized role of this activity in detoxification of peroxy compounds (Sun et al., 2016). Therefore, we used two different measurements of peroxide resistance, disc diffusion assays and bacterial growth curve analyses, to examine the response of wildtype and mutant bacteria grown under blue light versus in darkness to H_2_O_2_. Interestingly, we found that under the conditions of the disc diffusion assay, there was no measurable light-dependent difference in the response of 17978 wildtype bacteria to H_2_O_2_ challenge (**Figure 4A**). In the AB5075 strain of *A. baumannii*, KatG is the main enzyme responsible for H_2_O_2_ resistance measured using the same type of assay (Sun et al., 2016). To determine whether the same trend is observed in the 17978 strain, we further analyzed the peroxide resistance of the individual catalase mutant derivatives. Notably, as with wildtype bacteria, there was no light-dependent phenotype observed in any of the mutant strains (**Figure 4A**). The absence of both KatE and KatE2 caused a significant negative impact on the bacterial response to peroxide, although KatE2’s absence caused the greatest effect when compared to all other strains (**Figure 4A**). Importantly, complementation of the *katE2* gene in the cognate mutant background was successful in significantly decreasing the growth inhibition to levels comparable to those detected with the parental strain (**Supplementary Figure 4**). Remarkably, the absence of neither KatA nor KatG had a significant impact on peroxide resistance under the growth conditions used in the disc diffusion assay when compared with the parental strain (**Figure 4A**). Alternatively, the results of the growth curve analysis provided evidence of a significant, albeit slight, difference between the resistance of 17978 wildtype bacteria cultured under blue light compared to in darkness during exponential growth (**Figure 4B**). Compared to the wildtype strain at 8 h, all mutant strains exhibited a significant growth defect in the presence of H_2_O_2_, with 17978.*katE* exhibiting the greatest defect (**Figures 4C**–**F**). Furthermore, 17978.*katE* was the only strain that was completely unable to grow in the presence of 2.5 mM H_2_O_2_ across the entire 24 h time period during which growth was analyzed (**Supplementary Figure 5**; see supplemental figure for full growth data of all strains).

Taken together, these observations indicate that in the 17978 strain, 1) the catalase activity measured under our enzymatic assay conditions is light- and BlsA-regulated and mediated by KatE (**Figures 1**, **3**), and 2) of the four catalase homologs, KatE and KatE2 play the most significant roles in the breakdown of and overall resistance to H_2_O_2_ (**Figures 3**, **4A** and **Supplementary Figure 5**). Notably, not all catalase-related functions are light-regulated (compare **Figure 3** to **Figure 4A**). Additionally, KatG and KatA show only residual roles in catalase activity or general H_2_O_2_ resistance when cells are cultured at 24°C under shaking conditions (**Figures 4C, F** and **Supplementary Figure 5**).

### Investigation of the Role of BlsA as an Oxidative Response Regulator

Once we had investigated which of the catalase homologs and associated functions are regulated by light and BlsA, we set out to analyze the role of particular BlsA residues in the regulation of catalase activity. Specifically, we analyzed the impact of three different BlsA mutations on this regulation. Two of these mutations are substitution mutations of lysine residues at positions 144 and 145 to glutamic acid residues in both cases (K144E and K145E, respectively). The third mutation is a complete truncation of the last five amino acid residues of BlsA (Δ5). We have explored the effect of these three mutations on the capacity for BlsA to regulate surface-associated motility previously (Wood et al., 2019). Notably, both lysine residues are critical for BlsA’s role as a regulator of surface-associated motility, yet the five last BlsA residues are dispensable for said regulation.

**Figure 5:**
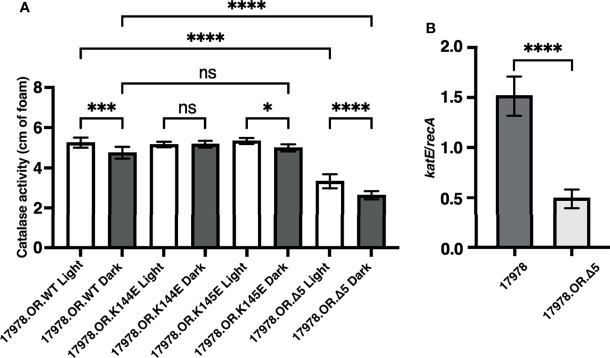
The role of key BlsA amino acid residues in KatE regulation. **(A)** Catalase activity of the isogenic 17978 *blsA* mutant complemented with pMU368 derivatives encoding either wildtype BlsA (WT), the BlsA amino acid substitution derivatives K144E and K145E, or the BlsA deletion derivative Δ5 was measured using culture samples representing equal numbers of bacteria that had been grown in SB under blue light or in darkness with shaking to exponential phase at 24°C. The catalase activity of each strain was analyzed using three independent biological replicates in technical triplicate (*n* = 9). Error bars represent the standard deviations of the data sets. Horizontal bars with symbols indicate results of ordinary one-way ANOVA with Sidak’s multiple comparisons *post-hoc* test (*P ≤ 0.05; ***P ≤ 0.001; ****P ≤ 0.0001; ns, not significantly different). **(B)** Expression of *katE* in the wildtype 17978 strain (17978) or the 17978 *blsA* mutant complemented with the pMU368 derivative encoding the BlsA deletion derivative Δ5 (17978.OR.Δ5). *katE* expression was normalized to that of the constitutively expressed reference gene *recA*. This transcriptional analysis was carried out for each strain using two independent biological replicates, the first analyzed in technical duplicate and the second analyzed in technical triplicate (*n* = 5). Error bars represent the standard deviation of the data sets. Horizontal bar with asterisks indicates the result of unpaired t-test analysis (****P ≤ 0.0001).

We used the assay described above to analyze the light-dependent catalase activity of the isogenic 17978 OR *blsA* insertion mutant (17978.OR) complemented with plasmids encoding the BlsA K144E, K145E, or Δ5 derivatives. This approach showed that OR cells producing the K145E BlsA protein (17978.OR.K145E) exhibited a differential light response similar to that displayed by cells producing the wildtype BlsA protein (17978.OR.WT), albeit not as statistically different between light and dark (**Figure 5A**). In contrast, such a differential response was not detected in bacteria producing the K144E protein (17978.OR.K144E). These results indicate that the K144 residue is important for BlsA’s ability to regulate KatE, which is not unexpected considering this residue is critical for BlsA’s regulation of surface-associated motility (Wood et al., 2019). What was unexpected was the catalase activity displayed by OR cells producing the Δ5 BlsA deletion derivative; this strain (17978.OR.Δ5) differentially produced catalase activity in response to illumination, but its overall catalase activity was significantly lower when compared with bacteria producing wildtype BlsA (**Figure 5A**). This observation is in contrast to results showing that the last 5 BlsA amino acid residues do not play a role in the expression of light-dependent BlsA-mediated 17978 surface motility responses (Wood et al., 2019). Importantly, this decrease in overall catalase activity cannot be attributed to a growth defect, as 17978.OR.Δ5 bacteria do not display any growth deficiency compared to wildtype (**Supplementary Figure 6**). Furthermore, the Δ5 deletion did not significantly affect the production of BlsA when overexpressed in *E. coli* or tested using *A. baumannii* total lysates prepared from wildtype and 17978.OR.Δ5 cells by western blotting as described before (Wood et al., 2019) (data not shown). Interestingly, when analyzed by reverse transcription quantitative PCR (RT-qPCR), bacteria producing the Δ5 deletion BlsA molecule exhibited significantly less *katE* expression compared to those producing the wildtype photoreceptor (**Figure 5B**).

**Figure 6:**
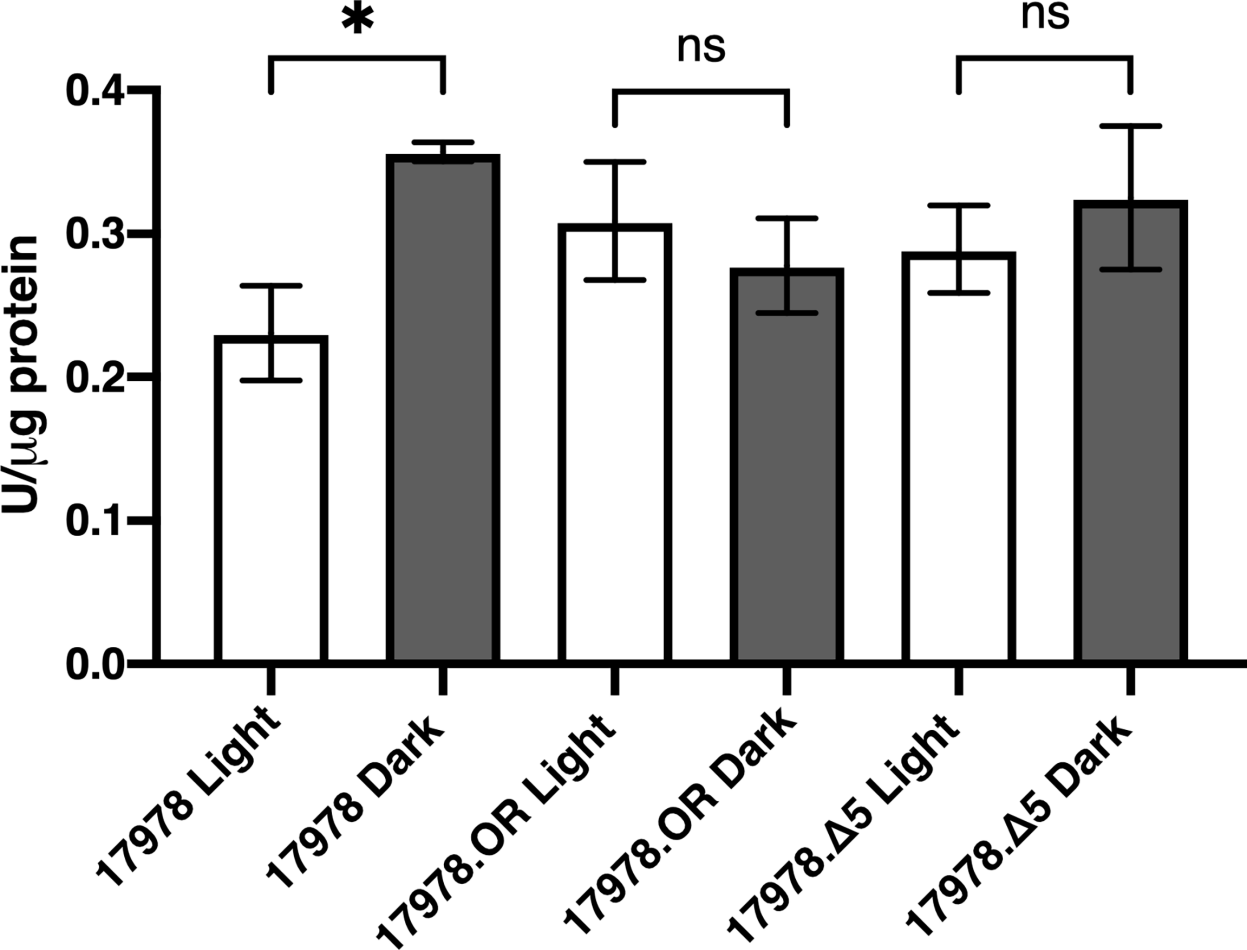
The role of BlsA in the regulation of superoxide dismutase activity. SOD levels of the 17978 wildtype and mutant strains were measured using culture samples representing equal numbers of bacteria that had been grown in SB under blue light or in darkness with shaking to exponential phase at 24°C. SOD activity was measured and normalized to total protein concentration for 2-3 independent biological replicates in technical triplicate. Error bars represent the standard deviations of the data sets. Horizontal bars with symbols indicate results of ordinary one-way ANOVA with Sidak’s multiple comparisons *post-hoc* test (*P ≤ 0.05; ns, not significantly different).

We hypothesized that, based on the data we obtained upon analyzing catalase activity in bacteria producing the Δ5 BlsA deletion derivative, BlsA’s last five residues may somehow play a role in eliciting an oxidative stress response, which then leads to an increase in *katE* expression and overall catalase activity that is independent of light. To test this possibility, the overall oxidative stress response of 17978 was examined using an assay to measure the production of SOD, a marker for such response. SOD catalyzes the dismutation of the superoxide radical $O_{2}^{-}$ to protect against both direct and indirect toxic damage. Specifically, superoxide is the primary ROS formed by photooxidation (Ziegelhoffer and Donohue, 2009). We analyzed the production of SOD in 17978 WT, 17978.OR, and 17978.OR.Δ5 bacteria cultured under blue light or in darkness. There was no significant difference in the oxidative stress responses of bacteria completely lacking BlsA compared to those producing the Δ5 BlsA deletion derivative (**Figure 6**). However, unexpectedly, the result of this analysis indicated that although catalase activity was higher when bacteria were cultured under blue light (**Figure 1**), SOD activity was downregulated in bacteria cultured under blue light (**Figure 6**). The fact that the absence of either the entire BlsA molecule or only its last five residues eliminated the light regulation of SOD activity observed in wildtype bacteria lends further importance to these residues that were, based on our previous study, thought to be dispensable for BlsA’s regulatory activity.

## Discussion

In this study, we set out to more thoroughly understand the light- and BlsA-dependent regulation of catalase activity in *A. baumannii*. Although the dependence of *katE* expression and overall catalase activity on light and BlsA has been investigated in *A. baumannii* previously (Muller et al., 2017), still outstanding was the specific scope of BlsA’s regulation of the total number of catalase enzymes, the corresponding impact on the physiology of the bacteria, and the importance of particular BlsA residues in said regulation. We were first able to show that catalase activity is a light- and BlsA-dependent process in a different *A. baumannii* strain than that in which the phenomenon was first observed (Muller et al., 2017) (**Figure 1**). Of note, in both strains, the regulation of catalase activity conferred by BlsA appears to be a fine-tuning activity mechanism rather than an on-off switch, which points to the necessity for catalase activity under either condition yet highlights the fact that a subtle, yet significant, upregulation of activity is important under blue light for a reason that is yet to be pinpointed.

Because *A. baumannii* encodes multiple catalase homologs including one catalase peroxidase (**Figure 2** and **Table 3**), and because of the previous evidence that two distinct catalases are light- and BlsA-regulated in *A. baumannii* (Muller et al., 2017), we set out to examine the impact of null catalase mutations on overall catalase activity and H_2_O_2_ resistance in response to light and, if applicable, BlsA. According to the results of the catalase activity assay in which we tested the enzymatic activity of catalase-deficient bacteria cultured under blue light and in darkness, we provide evidence that the catalase homolog that is regulated by BlsA and thus exhibits light-dependent activity is KatE, as inactivation of any of the other catalase genes did not affect the light-dependent regulation of catalase activity (**Figure 3A**). We also support this conclusion with zymogram analyses, the results of which identify the homologs whose activity are represented by the two protein bands. It is important to note that the light-dependent differences in activity exhibited by KatE and KatG when we analyzed in-gel catalase activity were not as apparent as what has been shown in the 19606 strain (Muller et al., 2017). We would argue that the catalase activity assay is more sensitive than the zymogram analysis and itself only demonstrates small but significant differences in catalase activity. Additionally, when comparing the results shown previously (Muller et al., 2017) and our data, the differences in strains analyzed and the overall growth conditions, including the use of different media and shaking *versus* stagnant culturing, may explain the less prominent light dependence of catalase activity exhibited in our zymogram analysis. Ultimately, the zymogram results support our finding from the catalase activity assay that KatE is light regulated. The significance of KatE in general catalase activity is also evident based on our H_2_O_2_ growth analysis, as the absence of KatE rendered bacteria completely unable to grow in the presence of H_2_O_2_ (**Figure 4D** and **Supplementary Figure 5**). The restoration of full catalase activity when *katE* was expressed from a complementing plasmid confirms that the *katE* mutation affected only one gene (**Figure 3B**). Our inability to restore light regulation may be based on a change in the overall number of KatE molecules within the cell after expression of *katE* from the complementing plasmid rather than from the chromosome, which in turn may have disrupted the fine-tuning of catalase activity that BlsA mediates. A similar phenomenon has indeed been observed when plasmid-based complementation assays were used to test the biological role of the 17978 *prpA* gene (Wood et al., 2018). Although it is speculative, a reason why *A. baumannii* may aim to regulate KatE in a light-dependent manner may involve an attempt to mitigate the photooxidation that is associated with light exposure (Orlandi et al., 2018).

Notably, the peroxide resistance of statically-cultured 17978 bacteria does not appear to be light regulated, as wildtype bacteria displayed comparable resistance responses independent of the presence or absence of light (**Figure 4A**). Still, upon analysis of the impact of each individual mutation on the capacity for bacteria to grow in the presence of H_2_O_2_, we discovered an interesting phenomenon: KatE2, a catalase that is not represented in the MDR *A. baumannii* strain AB5075, plays the most significant role in peroxide resistance under our experimental conditions. Indeed, the lack of KatE2 resulted in the highest sensitivity response among all tested strains when cultured statically (**Figure 4A**). This result was not unexpected when considered independently, as *katE2* expression is upregulated specifically by OxyR in response to H_2_O_2_ exposure (Juttukonda et al., 2019). However, we did not anticipate that the absence of KatE2 would have a greater impact on peroxide resistance compared to the absence of KatG (**Figure 4A**) because the KatG homolog in the AB5075 strain is the enzyme primarily responsible for H_2_O_2_ resistance in this strain, hypothetically due to its capacity as a peroxidase to detoxify peroxy compounds generated by H_2_O_2_ (Sun et al., 2016). We would propose a few explanations for this difference. Isolates 17978 and AB5075 are two distinct strains of *A. baumannii* with a key difference in the sets of catalase homologs that they possess: KatE2. Whereas OxyR is canonically known to upregulate *katG* in response to H_2_O_2_ (Michan et al., 1999), in the 17978 strain, OxyR upregulates *katE2* but not *katG* in response to H_2_O_2_ (Juttukonda et al., 2019). This regulation in and of itself points to the importance of KatE2 with regard to H_2_O_2_ resistance. In theory, the relative number of KatE2 molecules in the bacterial cell or its actual level of catalase activity may enable it to collectively decompose H_2_O_2_ at a great enough rate to lend it a relatively high significance in function, at least under static conditions. The fact that *Acinetobacter* sp. Ver3 encodes only two known catalases, both of which are related to KatE2, yet resists relatively high H_2_O_2_ levels (Sartorio et al., 2020), offers support for this position. However, additional investigation into KatE2’s function in 17978 is necessary to address this issue. Also worth mentioning is that our experimentation was carried out at 24°C, not the 37°C benchmark temperature typically used in studies involving the AB5075 strain. An exciting area of future study involves the question of whether the significance of KatE2 in H_2_O_2_ resistance is held at higher temperatures.

When BLASTed against non-redundant sequences, KatE2 was ubiquitous among *Acinetobacter* strains, including multiple clinical isolates, such as *A. baumannii* ABNIH5 and 1035119; however, the analysis of these strains has been mostly limited to genome sequencing. Undoubtedly, KatE2’s formal role in H_2_O_2_ resistance in *A. baumannii* has been somewhat overlooked because it is not encoded in the more well-studied AB5075 strain and otherwise has been misrepresented as KatE in a recent study (Juttukonda et al., 2019). Interestingly, ^AV3^KatE2 of the extremophile *Acinetobacter* Ver3 is a periplasmic catalase. The localization of this particular enzyme to the periplasm is dependent on an N-terminal signal peptide, which 17978’s KatE2 is also predicted to possess (data not shown). Although speculative, we expect that this potential localization influences the function of KatE2 in the 17978 strain. Lending further clues into the function of KatE2 is the discrepancy between the capacity for bacteria lacking KatE2 to grow in the conditions afforded by the disc diffusion assay compared to those of the growth curve assay (**Figures 4A, E**). Notably, each experiment presents a different physical constraint to *A. baumannii*: whereas the disc diffusion assays maintain bacteria in a static state, the catalase activity and growth curve assays involve shaking and therefore obligate movement. This observation indeed offers insight into the significance of multiple catalases for the different settings *A. baumannii* may encounter as a nonsocial pathogen that both persists on abiotic surfaces and colonizes and causes infections in a human host.

There was a slight but significant light-dependent phenotype when wildtype bacteria were in exponential phase in the presence of H_2_O_2_ (**Figure 4B**). Importantly, bacteria lacking either KatA, KatE, or KatG lost the light-related growth phenotype (**Figures 4C, D, F**), but KatE2’s absence did not make a difference with regard to this light regulation (**Figure 4E**). Based on the data we obtained indicating the overall significance and light-dependence of KatE in catalase activity (**Figure 3**) and its main role in peroxide resistance under shaking conditions (**Figures 4D** and **Supplementary Figure 5**), we propose that KatE is the enzyme responsible for the light-dependent growth phenotype. However, further analyses that are beyond the scope of this investigation are needed to answer this question definitively.

Along with providing evidence that BlsA regulates KatE specifically in a light-dependent manner, our data are also the first to indicate which BlsA residues are critical for this regulation. Based on the protein modeling and biological functional assays from our previous report on BlsA (Wood et al., 2019), we tested whether particular lysine residues are critical for BlsA’s regulation of catalase activity. The results of our experimentation demonstrate that lysine 144 is necessary for BlsA’s regulation of KatE (**Figure 5A**). Importantly, this residue is indispensable in BlsA’s regulation of surface-associated motility (Wood et al., 2019). Notably, the last five residues of BlsA are important for full catalase activity, but not the differential response to blue light (**Figure 5A**). This result was unexpected based on our previous data indicating that these five residues represent a non-functional region of the photoreceptor (Wood et al., 2019). It could be that although these residues are not involved in BlsA’s light-associated conformational change(s) nor in its interaction with the unknown mediator of surface-associated motility, they are important for the protein-protein interaction of BlsA with the regulator partner that controls *katE* expression such that their loss either strengthens or relaxes the interaction. Our finding that bacteria that produce a BlsA molecule which lacks its last five residues express significantly less *katE* compared to wildtype bacteria (**Figure 5B**) supports this hypothesis.

Based on the findings of our current study compared to those we reported recently (Wood et al., 2019), it could very well be that different residues of the BlsA molecule are necessary for its interaction with different binding partners. We expect that this question will be addressed more fully as additional BlsA binding partners are identified.

Our investigation into the role that SOD plays in response to blue light resulted in unexpected findings. The hypothesis that BlsA might somehow upregulate a photooxidative stress response, its five last amino acid residues being the means of said regulation, was not supported as the overall difference in SOD activity between bacteria producing wildtype BlsA and those producing Δ5 BlsA was not statistically significant (**Figure 6**). Our observation that SOD activity, a marker for oxidative stress response, was significantly higher in wildtype bacteria that had been cultured in dark conditions compared to under blue light was unexpected based on our catalase activity findings and the previous proposal that increased catalase activity in *A. baumannii* under blue light is in response to the increased oxidative stress presented by illumination (Muller et al., 2017). We have preliminary transcriptomic evidence that the two genes encoding SOD enzymes in 17978 cells are significantly downregulated in blue light compared to darkness in cells cultured under the same experimental conditions we describe in this report; therefore, our findings are not unfounded. Fur has been postulated to repress the expression of *sodB* based on a decreased expression of this gene in 17978 under low iron conditions. Based on the proposed interaction of BlsA with Fur in dark conditions specifically (Tuttobene et al., 2018) and Fur’s repression of *sodB* (Eijkelkamp et al., 2011), a Fur-mediated pathway may be the means by which BlsA controls SOD activity. Recent work involving the crystallization of BlsA, protein docking prediction experiments, and binding-affinity analyses has identified particular BlsA residues that are necessary for its interaction with Fur. Indeed, residues 101-110 are required for this interaction (Chitrakar et al., 2020). Although the significance of BlsA’s last five amino acid residues was not addressed in the study by Chitrakar et al. (2020), primarily because of their unsolvable nature during crystallography, further protein-protein interaction analyses would elucidate whether BlsA’s last five residues are necessary for stabilization of its interaction with the Fur transcriptional regulator.

In addition to interacting with Fur, BlsA interacts with BfmR (Chitrakar et al., 2020), a response regulator that was initially identified for its role in biofilm formation and bacterial cell morphology (Tomaras et al., 2008). Notably, as with Fur, the interaction of BlsA with BfmR is enhanced when BlsA is in its ground state, that is, when the protein is dark-adapted (Chitrakar et al., 2020). We postulate that the interaction of BlsA with BfmR is the key to BlsA’s regulation of KatE, as BfmR positively regulates KatE production in *A. baumannii* (Farrow et al., 2018). As a proof-of-concept, we demonstrated using the 19606 strain that, in the absence of BfmR, light-dependent KatE regulation is abolished, and overall catalase activity is significantly decreased (**Supplementary Figure 7**). We hypothesize that other BfmR-dependent *A. baumannii* functions, including pellicle formation, the expression of T6SS (Krasauskas et al., 2019), and biofilm formation (Tomaras et al., 2008), are effectively regulated by BlsA in a blue-light dependent manner (Mussi et al., 2010; Muller et al., 2017), based on the photoreceptor’s interaction with BfmR. It is worth reiterating that the enhanced interaction of dark-adapted BlsA with both BfmR, an activator of KatE, and Fur (Chitrakar et al., 2020), a repressor of SOD, fits well with our experimental data, *i.e.*, a relative decrease in KatE activity and a relative increase in SOD activity, both in darkness (**Figures 3**, **6**).

Taken together, the results of our investigation expand our understanding of how *A. baumannii* utilizes its various catalase enzymes and take a step further toward the complete understanding of the full scope of BlsA’s regulon. Furthermore, we believe the data obtained from this study provide a novel understanding of the impact of light and BlsA on the overall oxidative stress resistance mechanisms of *A. baumannii*.

## Data Availability Statement

The original contributions presented in the study are included in the article/**Supplementary Material**. Further inquiries can be directed to the corresponding author.

## Author Contributions

MS, HT, and LA contributed to the conceptualization and experimental design of the study. MS, HT, and LA performed experiments. MS, HT, and LA validated experimental design and provided experimental analysis. MS, HT, and LA contributed to drafting the manuscript. LA provided resources for experiments and supervision for the overall project. All authors approved the submitted version after participating in reading and revision of the final draft.

## Funding

This project was supported by National Institutes of Health Public Health grant R15GM117478-01 awarded to LA, as well as by Miami University research funds including a DUOS grant awarded to MS and HT. HT was supported by an Arnold and Mabel Beckman Foundation Research scholarship.

## Conflict of Interest

The authors declare that the research was conducted in the absence of any commercial or financial relationships that could be construed as a potential conflict of interest.

## Publisher’s Note

All claims expressed in this article are solely those of the authors and do not necessarily represent those of their affiliated organizations, or those of the publisher, the editors and the reviewers. Any product that may be evaluated in this article, or claim that may be made by its manufacturer, is not guaranteed or endorsed by the publisher.

## Supplementary Material

Supplementary Figure 1 Comparison of catalase homologs coded by the 17978 strain. Amino acid sequences of the 17978 KatA, KatE, and KatE2 proteins were compared using MUSCLE. Asterisks indicate identical residues, colons represent residues of groups of strongly similar properties, and periods identify residues of groups of weakly similar properties. The green, red, and orange residues represent conserved heme-binding, active and iron-binding sites, respectively.

Supplementary Figure 2 Comparison of *A. baumannii* and *E. coli* KatG homologs. Amino acid sequences of the KatG protein of the *A. baumannii* strains AB5075 and 17978 and the *E. coli* MG1655 strain were compared using MUSCLE. Asterisks indicate identical residues, colons represent residues of groups of strongly similar properties, and periods identify residues of groups of weakly similar properties. The conserved transition state stabilizer, proton acceptor, and iron-binding sites are highlighted in blue, red, and orange, respectively. Residues of the Trp-Tyr-Met interaction necessary for catalase activity but not peroxidase activity of the *E. coli* KatG are highlighted in green.

Supplementary Figure 3 Growth of catalase mutant bacteria compared to the wildtype strain. The growth of the four 17978 isogenic catalase mutant derivatives in the presence of blue light or in darkness was compared to that of wild type. Bacteria were grown in the same conditions used for catalase activity assays. These data are representative of two separate experiments using independent biological samples each time.

Supplementary Figure 4 Peroxide resistance of the *katE2* complemented strain. The impact of light on the peroxide resistance of 17978 wildtype cells and the isogenic *katE2* mutant strain (17978.*katE2*) with its complemented derivative (17978.*katE2*WT) was measured using a disc diffusion assay. The resistance of bacteria grown on SA plates in the presence of sterile filter disks impregnated with 10 µL of 9.8 M (30%) H_2_O_2_ was analyzed by measuring diameters of growth inhibition halos after overnight incubation at 24°C in the presence or absence of illumination. Shown are the average diameters of inhibition from three independent experiments using different biological samples with each analyzed in triplicate (*n* = 3). Error bars represent the standard deviations of the data sets. Horizontal bars with symbols indicate results of ordinary one-way ANOVA with Sidak’s multiple comparisons *post-hoc* test (****P* ≤ 0.001; *****P* ≤ 0.0001).

Supplementary Figure 5 Growth of 17978 wildtype and catalase mutant bacteria in the presence of H_2_O_2_. The growth of the *kat* mutants in the presence of H_2_O_2_ under blue light or in darkness was compared to that of wildtype in the same condition. Bacteria were grown in SB supplemented with 2.5 mM H_2_O_2_ at 24°C with shaking, and optical density was measured using a spectrophotometer over time. These data are the average of at least two separate experiments using independent biological samples each time with error bars representing the standard deviations of the data sets.

Supplementary Figure 6 Growth of 17978.OR.Δ5 bacteria compared to wildtype. Bacteria were grown in the same conditions used for catalase activity assays. These data are representative of two separate experiments using independent biological samples each time.

Supplementary Figure 7 The effect of the absence of BfmR on catalase activity of the type strain *A. baumannii* ATCC 19606^T^. Catalase activity was measured using culture samples representing equal numbers of bacteria that had been grown in SB under blue light or in darkness with shaking to exponential phase at 24°C. The catalase activity of each strain was analyzed using three independent biological replicates in technical triplicate (*n* = 9). Error bars represent the standard deviations of the data sets. Horizontal bars with symbols indicate results of ordinary one-way ANOVA with Sidak’s multiple comparisons *post-hoc* test (****P* ≤ 0.001; *****P* ≤ 0.0001; ns, not significantly different).
