# Supplementary figures and images for: The Influence of Blue Light and the BlsA Photoreceptor on the Oxidative Stress Resistance Mechanisms of Acinetobacter baumannii
Source: Front Cell Infect Microbiol. 2022 Mar 24;12:856953. doi: 10.3389/fcimb.2022.856953 (PMC8987720; doi:10.3389/fcimb.2022.856953)

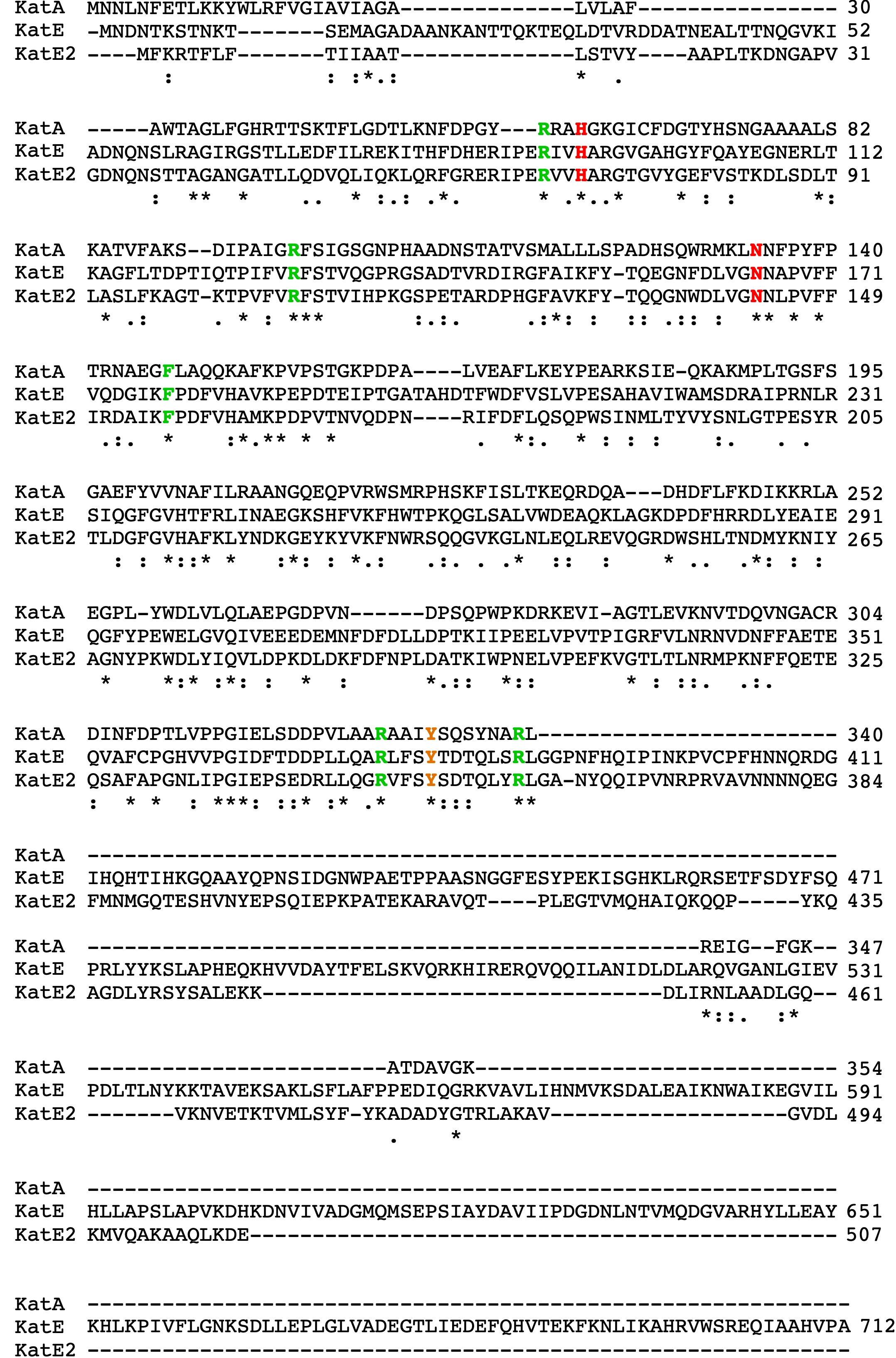

Supplement: Supplementary Figure 1 — Comparison of catalase homologs coded by the 17978 strain. Amino acid sequences of the 17978 KatA, KatE, and KatE2 proteins were compared using MUSCLE. Asterisks indicate identical residues, colons represent residues of groups of strongly similar properties, and periods identify residues of groups of weakly similar properties. The green, red, and orange residues represent conserved heme-binding, active and iron-binding sites, respectively. [file Image_1.tif]

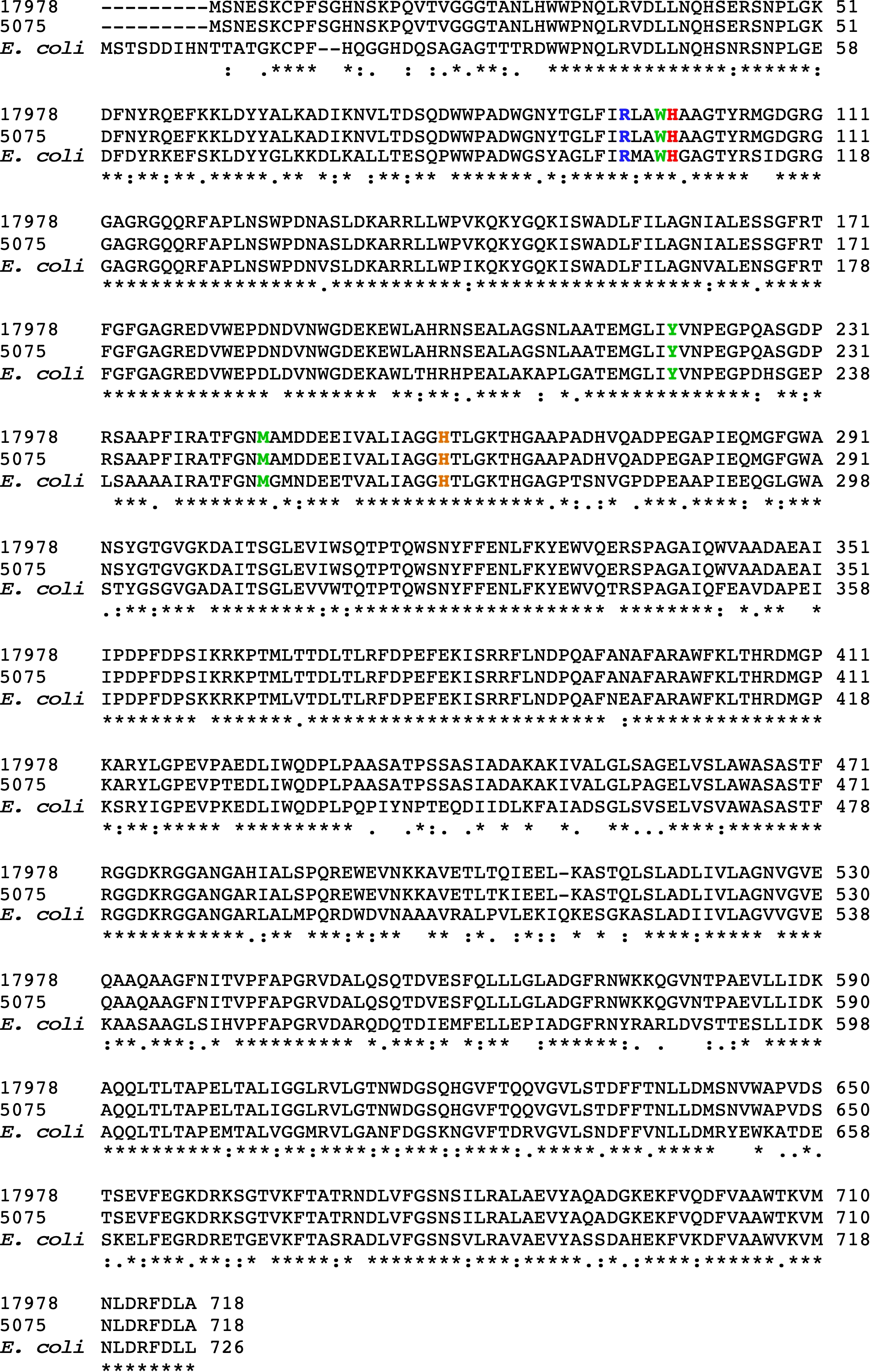

Supplement: Supplementary Figure 2 — Comparison of A. baumannii and E. coli KatG homologs. Amino acid sequences of the KatG protein of the A. baumannii strains AB5075 and 17978 and the E. coli MG1655 strain were compared using MUSCLE. Asterisks indicate identical residues, colons represent residues of groups of strongly similar properties, and periods identify residues of groups of weakly similar properties. The conserved transition state stabilizer, proton acceptor, and iron-binding sites are highlighted in blue, red, and orange, respectively. Residues of the Trp-Tyr-Met interaction necessary for catalase activity but not peroxidase activity of the E. coli KatG are highlighted in green. [file Image_2.tif]

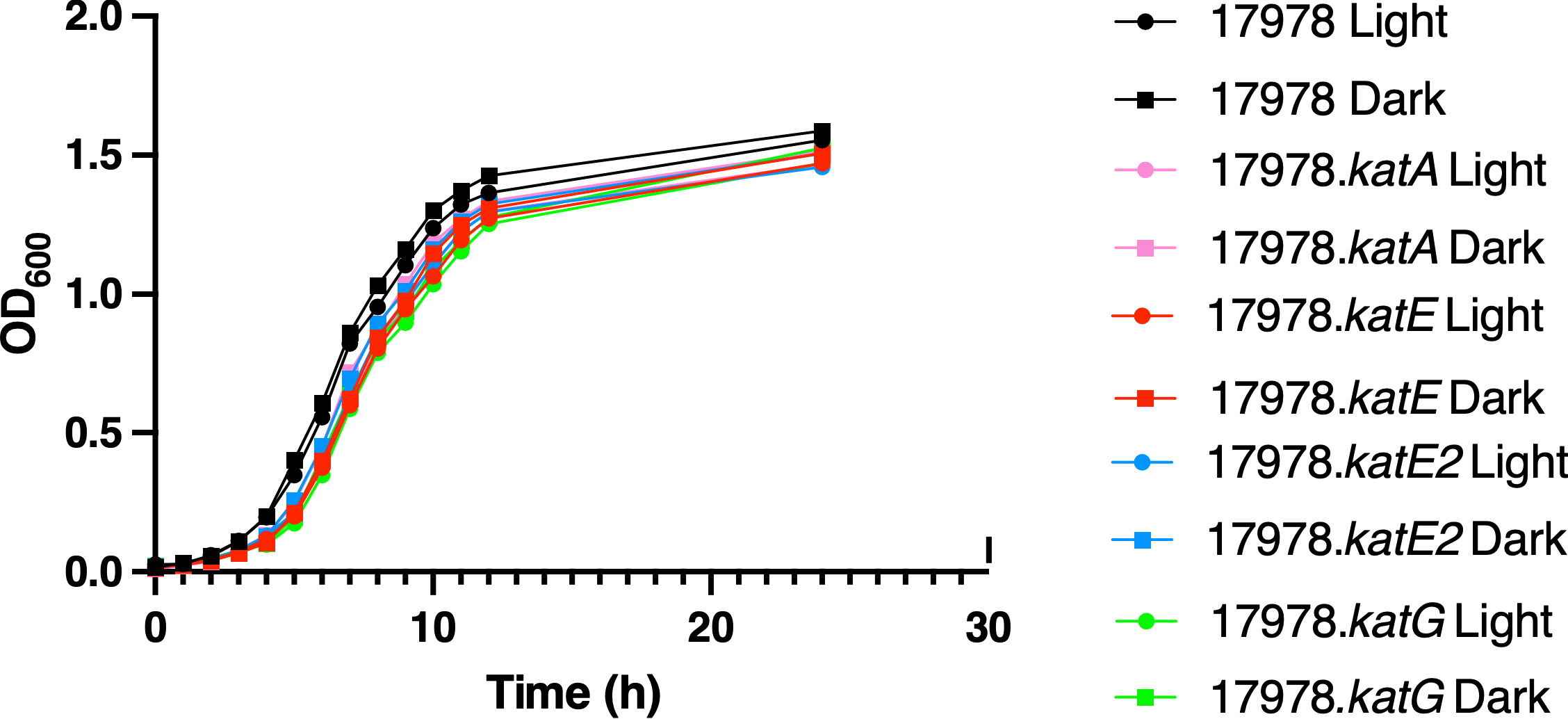

Supplement: Supplementary Figure 3 — Growth of catalase mutant bacteria compared to the wildtype strain. The growth of the four 17978 isogenic catalase mutant derivatives in the presence of blue light or in darkness was compared to that of wild type. Bacteria were grown in the same conditions used for catalase activity assays. These data are representative of two separate experiments using independent biological samples each time. [file Image_3.tif]

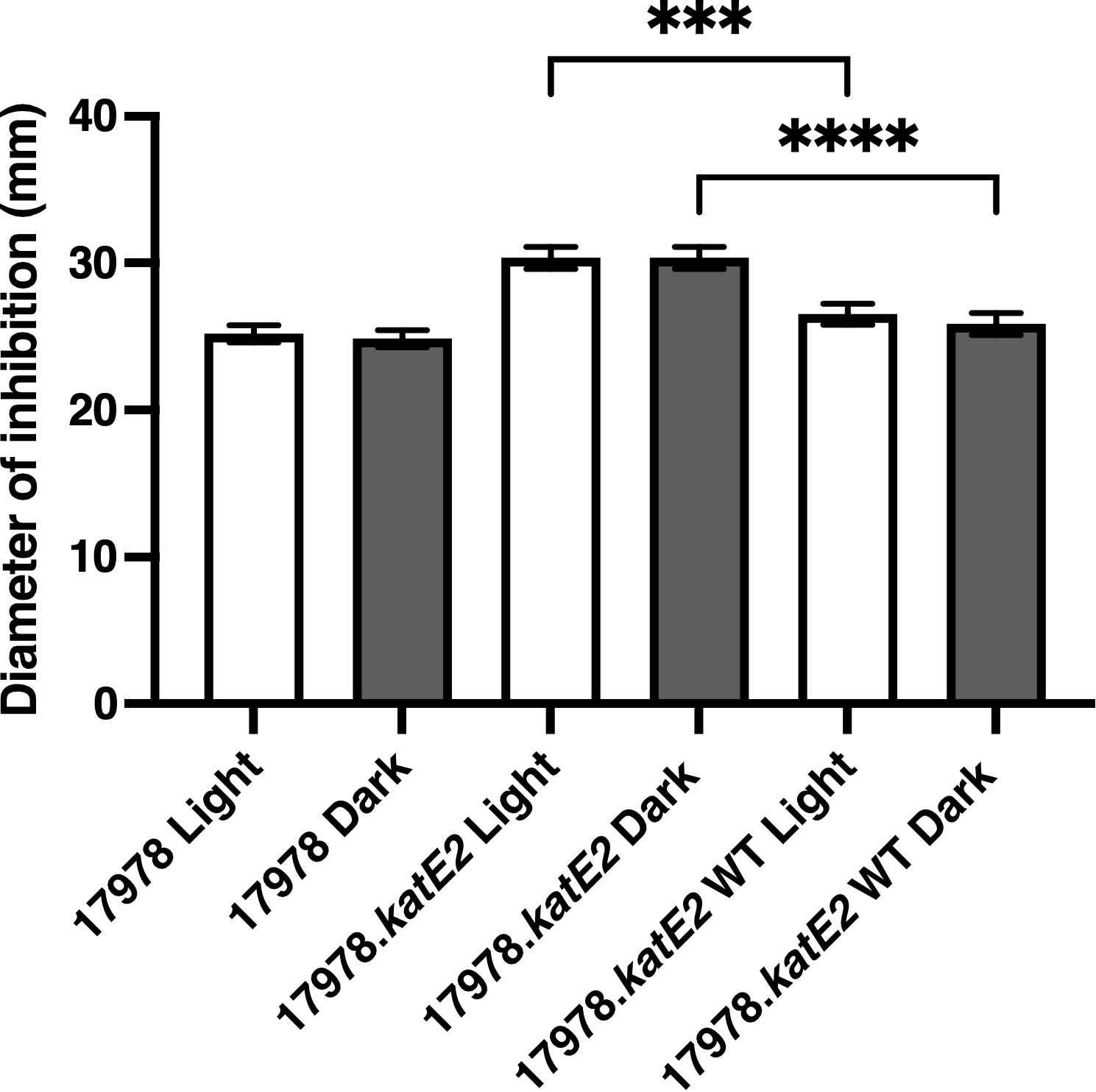

Supplement: Supplementary Figure 4 — Peroxide resistance of the katE2 complemented strain. The impact of light on the peroxide resistance of 17978 wildtype cells and the isogenic katE2 mutant strain (17978.katE2) with its complemented derivative (17978.katE2WT) was measured using a disc diffusion assay. The resistance of bacteria grown on SA plates in the presence of sterile filter disks impregnated with 10 µL of 9.8 M (30%) H2O2 was analyzed by measuring diameters of growth inhibition halos after overnight incubation at 24°C in the presence or absence of illumination. Shown are the average diameters of inhibition from three independent experiments using different biological samples with each analyzed in triplicate (n = 3). Error bars represent the standard deviations of the data sets. Horizontal bars with symbols indicate results of ordinary one-way ANOVA with Sidak’s multiple comparisons post-hoc test (***P ≤ 0.001; ****P ≤ 0.0001). [file Image_4.tif]

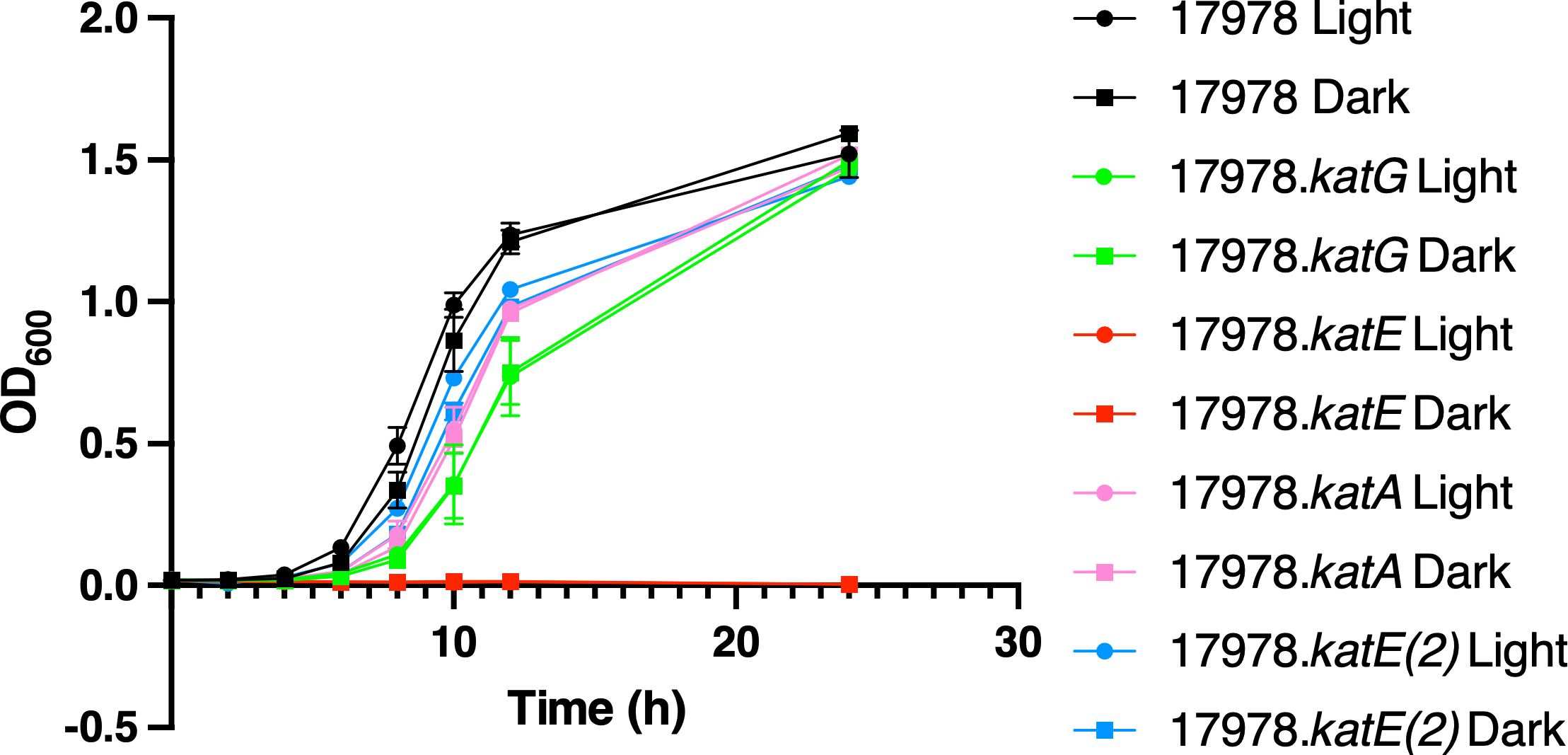

Supplement: Supplementary Figure 5 — Growth of 17978 wildtype and catalase mutant bacteria in the presence of H2O2. The growth of the kat mutants in the presence of H2O2 under blue light or in darkness was compared to that of wildtype in the same condition. Bacteria were grown in SB supplemented with 2.5 mM H2O2 at 24°C with shaking, and optical density was measured using a spectrophotometer over time. These data are the average of at least two separate experiments using independent biological samples each time with error bars representing the standard deviations of the data sets. [file Image_5.tif]

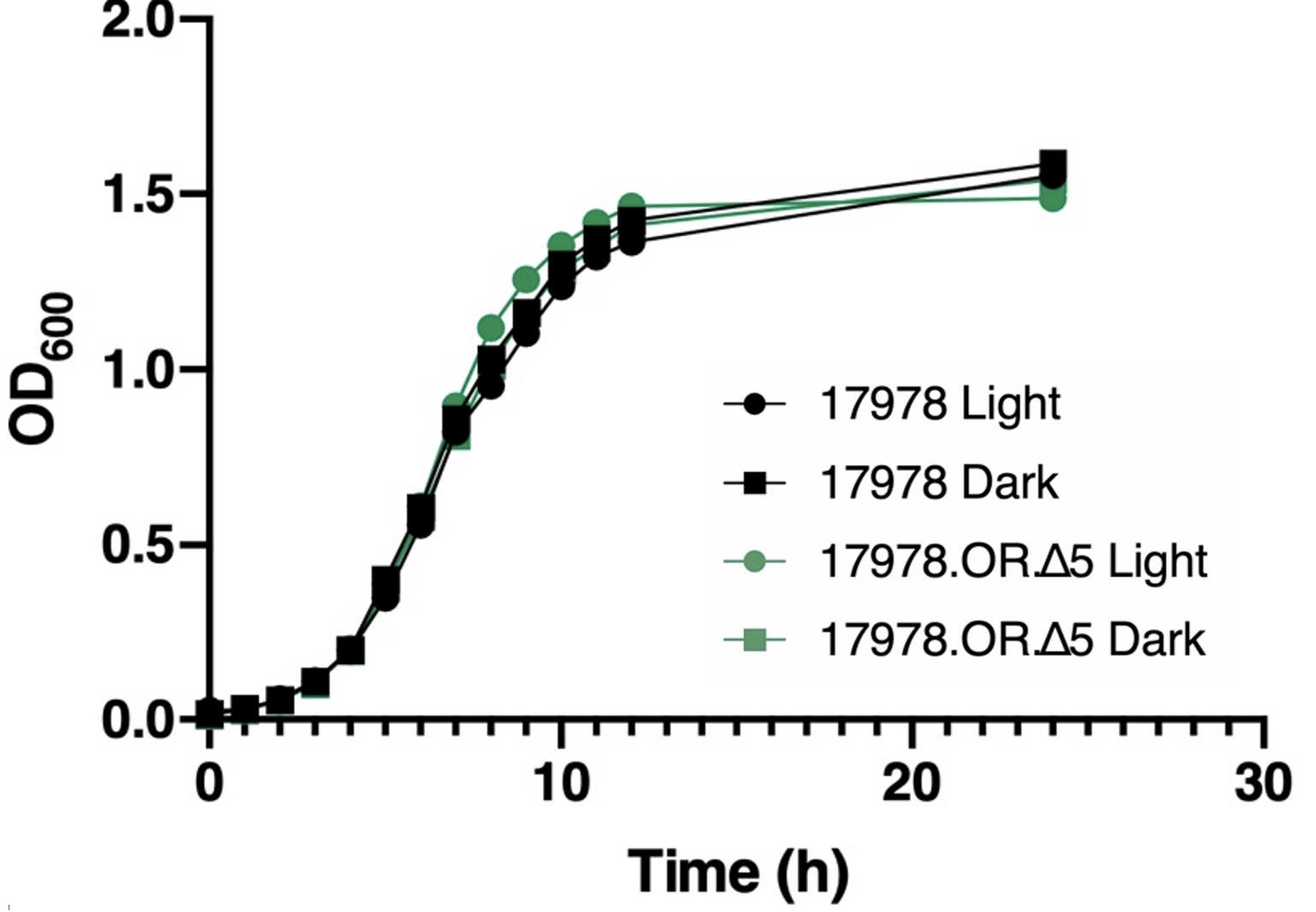

Supplement: Supplementary Figure 6 — Growth of 17978.OR.Δ5 bacteria compared to wildtype. Bacteria were grown in the same conditions used for catalase activity assays. These data are representative of two separate experiments using independent biological samples each time. [file Image_6.tif]

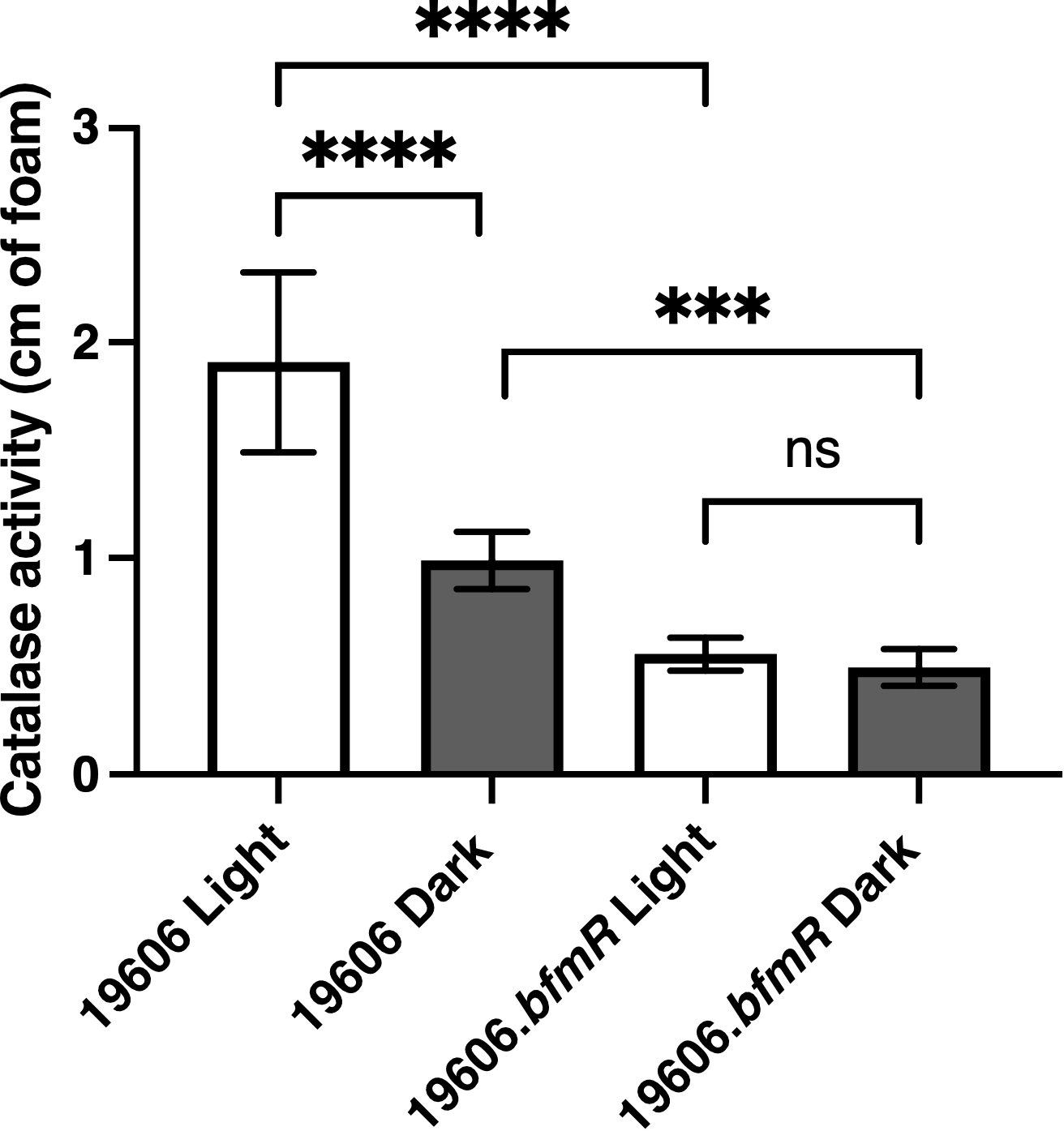

Supplement: Supplementary Figure 7 — The effect of the absence of BfmR on catalase activity of the type strain A. baumannii ATCC 19606T. Catalase activity was measured using culture samples representing equal numbers of bacteria that had been grown in SB under blue light or in darkness with shaking to exponential phase at 24°C. The catalase activity of each strain was analyzed using three independent biological replicates in technical triplicate (n = 9). Error bars represent the standard deviations of the data sets. Horizontal bars with symbols indicate results of ordinary one-way ANOVA with Sidak’s multiple comparisons post-hoc test (***P ≤ 0.001; ****P ≤ 0.0001; ns, not significantly different). [file Image_7.tif]
